# Computational Design and Expression of Headless Influenza Hemagglutinin Antigens Toward a Modular Universal Nanoparticle Vaccine

**DOI:** 10.3390/antib15040064

**Published:** 2026-07-27

**Authors:** Victor Ovchinnikov, Martin Karplus

**Affiliations:** 1 Department of Chemistry and Chemical Biology, Harvard University, Cambridge, MA 02138, USA; 2 Laboratoire de Chimie Biophysique, Institut de Science et d’Ingénierie Supramoléculaires, Université de Strasbourg, 67000 Strasbourg, France

**Keywords:** immunization, universal antigen design, simulation, hemagglutinin

## Abstract

Background: The elicitation of broadly neutralizing antibodies against conserved antigenic epitopes has been a focus of universal vaccine research. To facilitate immunofocusing on the conserved influenza hemagglutinin (HA) stalk, we designed headless trimeric antigens, initially focusing on subtypes H1, H3, and H5, and subsequently expanding to eight additional HA subtypes. Results: The designs were first evaluated in silico; they were predicted to fold correctly by AlphaFold2, and remained stable in molecular dynamics simulations in isolation, as well as bound to a broadly neutralizing antibody. The antigens expressed in HEK293-derived cells in high yields, and eluted predominantly as trimers in SEC-HPLC. Future work will explore the use of mosaic nanoparticles decorated with headless antigens of various subtypes for the optimal elicitation of broadly neutralizing anti-influenza antibodies. Overall, our study advances the use of headless HA trimers as modular antigens toward a universal influenza vaccine.

## 1. Introduction

Influenza remains a significant cause of morbidity and mortality around the world [1]. Mutations in the surface proteins hemagglutinin (HA) and neuraminidase, compounded by high viral transmission rates and evolutionary pressure exerted by vaccine-induced antibodies, cause the emergence of escape variants, against which existing antibody-based immunity induced by prior immunizations is ineffective. Thus, seasonal flu vaccines typically need to be readministered on a yearly basis. Further, the time lag in producing the current protein-based seasonal vaccines often results in a low level of protection (10–60%) [2]. Despite the high overall mutation rate of influenza genomes [3], surface epitopes that are relatively well conserved between strains have been discovered. In the case of the influenza HA, several of them, though not all [4], are located on the stalk or base of the HA [5,6]. Antibodies (Abs) that bind these epitopes [6,7,8,9] are important for universal flu vaccine research [10], since, if elicited by immunization, they could provide protection against many different viral strains. Such broadly neutralizing Abs (bnAbs) have been isolated in response to natural infection or vaccination [6], and some have been engineered to have very high breadth [11], providing simultaneous protection against phylogenetically distant group I and II HAs [11,12,13]. BnAbs have also shown heterosubtypic protection in passive immunizations [6], demonstrating their ability to provide broad-based immunity. Statistically, the presence of bnAbs correlates inversely with symptomatic disease [14]. BnAbs against HAs are more commonly found in older individuals, suggesting that their elicitation is rare in vaccinations or natural infections, relative to strain-specific Abs. Indeed, the conserved epitopes targeted by many bnAbs are immunosubdominant, i.e., they are not the primary targets of immune responses. Many different reasons for immunosubdominance have been proposed, e.g., the negative selection of autoreactive B-cells [15], the low frequency of precursor Abs able to bind a given conserved epitope [16], glycan shielding [17], preexisting immune imprinting [16,18], poor epitope accessibility [19], a lack of compatible MHCII (major histocompatibility complex of type 2) epitopes [20], and an abundance of distracting epitopes [19,21,22], such as the large size of the HA head, relative to the sialic acid binding site. While exploring the causes of immunosubdominance is beyond the scope of this paper, it is clear that universal influenza vaccines aiming to elicit bnAbs must include strategies to overcome it. Typically, universal vaccine candidates present multiple epitopes to the immune system, in the form of chimeric antigens composed of different epitopes [23], as multi-antigen cocktails [22,24], or via the conjugation of different antigens to nanoparticles (NPs) [25,26,27]. Nanoparticle vaccines are of special interest because they have been shown to direct the immune response to conserved epitopes [25,26,28,29,30]. For example, Kanekiyo et al. [25] co-displayed HA receptor binding domains (RBDs) from different influenza strains on NPs, and found that the resulting antibodies had greater breadth than those elicited by NPs of homogeneous compositions. The increased breadth was attributed to an avidity advantage [25], which was later modeled computationally [22,31]. In addition, because the HA head is a source of variable immunodominant epitopes, a common design approach is to produce “headless” antigens that only contain the HA stalk, using suitable structural stabilization [29,32,33,34,35,36]. Such antigens, based on HAs from H1 [34,35] and H5 [36] subtypes, conferred protection group I viruses, but also protected mice from mortality in an H3 (group II) challenge [36]. Headless influenza antigens have previously been presented on NPs, including subtypes H1 [35], H3 [29], H7 and H10 [37], which were produced by genetically fusing the antigens to *H. pylori* ferritin. As an alternative to fusion to NP precursors, full-length influenza HAs have been genetically fused to 12-residue SpyTag peptides [27], followed by covalent conjugation to NPs bearing the SpyCatcher protein [38]. With this method, Cohen et al. [27] conjugated up to eight different full-length HAs to mi3 NPs [39]. Mice immunized with either homotypic or mosaic NPs elicited strong immune responses, demonstrating that the mi3 NPs are potential alternatives to seasonal flu vaccines. However, the apparent lack of increased breadth in sera obtained from mosaic NP immunizations vs. homotypic NP mixtures suggested that additional modifications would be needed to produce a broadly reactive universal vaccine, such as the removal of immunodominant epitopes from full-length HAs. Here, we extend previous approaches [29,35,37] by designing computationally stable soluble headless influenza antigens of subtypes H1, H3, and H5. An important aspect of our method is the use of a new trimerization domain from collagens of type XV (CXV) [40], which appears to facilitate more stable antigen trimers, relative to the commonly used foldon trimerization domain from the T4 bacteriophage [41]. Further, we demonstrate high-yield mammalian expression of headless HAs from eight additional subtypes, which cover the landscape of influenza strains, and could therefore form the basis of a universal NP vaccine candidate.

## 2. Methods

### 2.1. Antigen Design

Headless HA designs were created as described by Yassine et al. [35] and Corbett et al. [29] for HAs in groups I and II, respectively, except that the collagen type XV (multiplexin) [40,42,43] was used in place of the T4 foldon to facilitate trimerization. Coordinate manipulation and minimization was performed using the program CHARMM (v.47b1) [44]. The completed designed sequences were submitted to a local installation of AlphaFold v.2 multimer for structure prediction, requesting five trimeric model predictions for each sequence. The design sequences are aligned in Tables S1 and S3.

### 2.2. MD Simulations

Initial coordinates for molecular dynamics (MD) simulations were prepared starting from the models computed with AlphaFold2 [45]. Simulation input files were created using the programs Matlab (R2021b) [46] and CHARMM [44]. To increase simulation speed and reduce storage requirements, we performed all MD simulations using a quasi-equilibrium solvation shell model [47], in which the protein atoms were surrounded by a thin layer of solvent, with evaporation prevented using restraint potentials, written as a plugin for the MD library OpenMM [48]. The solvation model has been validated in unbiased MD as well as in free energy simulations, and has been found to introduce relatively minor artifacts, in the form of a slight but controllable departure from equilibrium dynamics, and increased solvent pressure on the protein [47]. Comparisons of potentials of mean force (PMFs) profiles of antigen–antibody separation produced with the shell model vs. the periodic solvent box showed that the differences between them were small [49]. Sodium and chloride ions were added to the solvent shell to achieve a total ion concentration of 150 mM. The use of a solvent shell of 8.5 Å thickness resulted in total atom counts in the range 27 K–31 K for the hemagglutinin (HA) stalk trimers, with the exact count dependent on the design simulated. For the H3 HA trimer in the complex with the FI6V3 antibody Fabs, the total number of atoms was 47,776. A density controller was used to maintain solvent density in the outer solvation shell at ≃1 g/mL by dynamically adjusting the solvent layer thickness, and a rigid-body restraint with force constant k = 10 kcal/mol/Å^2^ was used to prevent rigid-body motion of the protein complexes [47]. All solvated systems were simulated using the OpenMM (v.7.3) [48] library via the Python (v.3.13) interface with the CHARMM36m energy function [50] in TIP3 water [51]. Other MD simulation parameters included reaction-field electrostatics [52], a 9 Å nonbonded cutoff for Lennard-Jones interactions, and hydrogen-mass repartitioning (HMR), which transfers 3 a.m.u. to every hydrogen from the parent heavy atom, and allows using a 4 fs simulation step. Covalent bonds to hydrogen were kept rigid, as were the water molecules, and the equations of motion were integrated using a Langevin dynamics integrator at 300 K with the friction coefficient set to 1 ps^−1^. The MD simulation speeds were ∼500 ns/day for the HA trimers and ∼300 ns/day for the H3 trimer bound to the FI6V3 Fab, using a workstation with an NVIDIA RTX 2080Ti GPU and a Ryzen 7900X CPU. For the simulations of HA trimers, the first 4 ns of each simulation were performed with RMSD restraints applied to all $C_{\alpha }$ protein atoms with a force constant of 1 kcal/mol/Å^2^, to keep the simulation structure near the initial structure as part of equilibration. For the simulation of H3 bound to FI6V3, the restraint force constant was lowered to 0.1 kcal/molÅ^2^, and the equilibration time was extended to 40ns to achieve a more gentle equilibration of the docked complex. Visualization of structures and calculation of root-mean-square distances (RMSDs) were performed using Visual Molecular Dynamics [53], and Matlab [46], respectively.

### 2.3. Preparation of Antigens

For each design, the target DNA sequence was designed from the corresponding amino acid sequence using Genscript’s codon optimization tool (https://www.genscript.com/gensmart-free-gene-codon-optimization.html; accessed 20 February 2025) and subcloned into the pcDNA3.4 vector with cloning sites EcoRI(G/AATTC)/HindIII(A/AGCTT). The signal sequence MGWSCIILFLVATATGVHS was used with all protein sequences, and a hexa-His tag was added at the end. Transfection-grade plasmid was prepared for HD 293F cell expression using a Genscript maxi-prep kit (Piscataway, NJ, USA). High-density (HD) 293F cells were grown in the freestyle expression medium, in flasks at 37 °C with 8% CO_2_ inside an orbital shaker. On the day before transfection, cells were seeded in separate flasks. The following day, DNA and a transfection reagent were mixed and added to the flasks containing seeded cells. Recombinant plasmids encoding target proteins were transiently transfected into suspensions of HD 293F cell cultures. Cell culture supernatants were collected on day 6 and were used for subsequent purification. The cell culture broth was centrifuged and filtered, and the filtered supernatant was loaded onto the HisTrap FF Crude column. After washing and elution, the eluted fractions were pooled and buffer-exchanged to the final formulation buffer (PBS at pH 7.2). The purified protein was analyzed by SDS-PAGE and size-exclusion/high-performance liquid chromatography (SEC-HPLC) to determine molecular weight and purity. Protein concentration was measured by UV absorbance at 280 nm.

### 2.4. Principal Component Analysis of Influenza Strain Landscape

To visualize the landscape of influenza hemagglutinin (HA) sequences, we performed principal component analysis (PCA) on the embedded protein coordinates defined by Atchley et al. [54], as described before [55].

Briefly, sequences of avian, swine and human influenza type A HA proteins spanning the years 1918–2019 and subtypes 1–18 were downloaded from the NIH influenza research database [56] and were clustered and sampled so that no two retained sequences were more than 95% identical. Each sequence was pairwise aligned to the H1 sequence from virus A/New_York/1105/2008 with accession number AHL90133.1 (www.gisaid.org, accessed 25 February 2025), and the residues corresponding to the head domain (after VTVTHSVNLLEN and through VHPVTIGECP) and transmembrane domain (after GVYQILA) were deleted. The resulting stalk-only sequences were multiply aligned, and each residue was associated with a quintuplet coordinate (5D vector space) of Atchley et al. [54], which was found to give a good visual separation of the HA subtypes on the 3D landscape (see Section 4).

The covariance matrix of the embedded sequences was computed as

(1) $$C_{jk}^{pq}=\langle \left(x_{ij}^{p}-{\langle x_{ij}^{p}\rangle }_{i}\right)\langle {(x_{ik}^{q}-{\langle x_{ik}^{q}\rangle }_{i}\rangle }_{i},$$

where the angle brackets represent averages over sequences (rows in the MSA), which are indexed by *i*; *j* and *k* denote residue positions, and *p* and *q* denote embedded coordinate components. The covariance matrix was diagonalized to yield the diagonal eigenvalue (EV) matrix Ω and eigenvectors (principal components; PCs) *V*,

(2) $$C=V\Omega V^{-1}.$$

The coordinate projection of any sequence *s* onto any PC vector, e.g., ${\mathrm{PC}}_{l}^{q}$, is computed as

(3) $$PC_{l}^{q}=\sum_{j=1}^{N_{res}}\sum_{p=1}^{5}V_{lj}^{qp}\left(x{\left(s\right)}_{j}^{p}-{\langle x_{ij}^{p}\rangle }_{i}\right),$$

where x(s) are the embedded coordinates of *s* (aligned with the MSA) [54,55]. The double index on $PC_{l}^{q}$ is retained for simplicity; in practice, we sort the PCs in the order of decreasing EVs, and plot projections onto the three PCs corresponding to the highest EVs. The PCA was performed in Matlab [46].

## 3. Results

**Figure 1:**
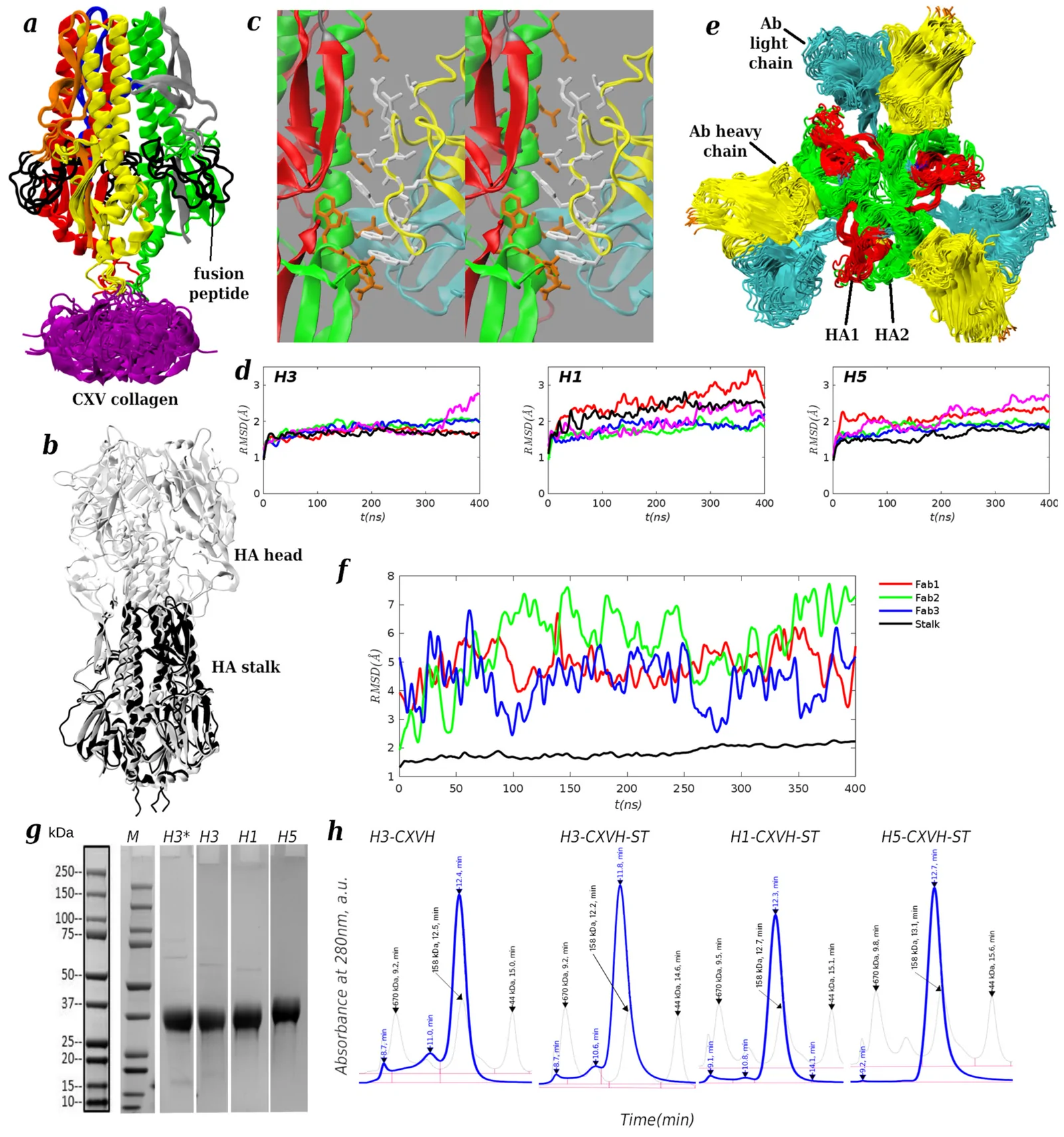
Characterization of headless influenza designs. (**a**) Superposition of five structures of the H3 design predicted by AlphaFold2(AF2); the solvent-exposed fusion peptide is drawn as a black coil, and CXV multiplexin trimers are in purple; (**b**) superposition of the first AF2 predicted structure of the H3 design onto the full-length HA from PDB 3ZTJ; (**c**) wall-eyed stereo view of the FI6V3/H3 interface; the Fab heavy and light chains are in yellow and cyan, respectively, and the HA1 and HA2 subunits are in red and green, respectively; (**d**) RMS deviations of simulation structures from the corresponding initial (AF2-predicted) structure (RMSD excludes CXV and fusion peptide); differently-colored traces in each subpanel (red, green, blue, magenta, black) correspond to the five predicted structures; (**e**) snapshots from 400 ns MD simulations of the FI6V3 antibody Fabs from PDB 3ZTJ docked into the first H3 AF2-predicted structure; colors are as in (**c**); (**f**) RMS deviations of Fabs and the HA stalk from a 400 ns MD simulation; the simulation structures were superposed by aligning to the first AF2-predicted structure of H3. (**g**) Reduced SDS-PAGE and (**h**) SEC-HPLC chromatograms for designs H3-CXVH, H3-CXVH-ST, H1-CXV-ST, and H5-CXVH-ST in Table 1. In panel (**g**), design H3-CXVH (without the SpyTag) is denoted by H3* for brevity; the remaining designs include the SpyTag. Reduced and non-reduced SDS-PAGE are shown for individual designs in Figure S1. SDS-PAGE and the chromatogram for design H1-CXVZ-ST are shown in Figure S2.

To produce stable headless antigen trimers in a high yield, we modified the designs of Yassine et al. [35] and Corbett et al. [29], who described a series of iterative linker optimizations to produce HA stalk trimers of subtypes H1 (group I) and H3/H7 (group II), respectively. Although the authors were able to produce a foldon-stabilized H1 headless design, the production of a headless H3 required replacing the foldon by genetic fusion to *H. pylori* ferritin [29]. We hypothesized that the ferritin provided increased trimer stabilization of the headless stalks, vs. the smaller (27-residue) T4 foldon, and set out to test collagens as alternative trimerization domains. While there are several types of domains that initiate or facilitate trimerization of collagen fibrils [40], we focused specifically on type XV collagens (multiplexins), because they are relatively small, a monomer being 54 amino acid residues long, form stable trimers at picomolar concentrations, have a crystal structure available to facilitate structure-based design, and express readily in mammalian cells [43]. In the past, producing stable headless HA antigens required at least several design iterations [33]. While the H1 design based on the A/New Caledonia/20/1999 strain was straightforward, requiring six iterations [35], the H3 and H7 designs of Corbett et al. [29] apparently required more than two hundred mutants, as well as fusion to ferritin, to achieve high expression levels. To increase the probability of success prior to expression, we first predicted the structures of our CXV-stabilized designs using AlphaFold2 [45]. Five independent predictions were generated for each of the three design subtypes: H1, H3 and H5. In each case, all five showed well-folded trimers. The H3 design is shown in isolation in Figure 1a, in superposition on the full-length H3 HA from PDB 3ZTJ in Figure 1b, and docked to the bnAb FI6V3 in Figure 1c. The five models for each subtype were similar, with the maximum backbone root-mean-square deviation (RMSD) between any two of them, excluding the trimerization domain and the fusion peptide (black coil in Figure 1a), of 1.93 Å, 1.47 Å, and 0.82 Å, for H1, H3 and H5, respectively. The CXV trimerization domain was excluded from the RMSD because it is connected to the HA stalk using a flexible linker, and is therefore expected to have many compatible orientations relative to the stalk. Aside from the flexibility in this linker region, the models differed in the precise positioning of the fusion peptide that connects the HA1 and HA2 subunits in the hemagglutinin precursor HA0 [57]. The differences are suggestive of loop flexibility, which is consistent with the structure of HA0 [57], in which the peptide is exposed to the solvent, as well as with the existing X-ray structures of headless H1 [34,35], in which the peptide is partly unresolved. To investigate the overall stability and flexibility of the designed structures more extensively, we simulated all of them by molecular dynamics (MD) simulation (see Section 2). For most of the structures, the backbone RMSD from the corresponding AlphaFold2 prediction was around 2 Å throughout the duration of the simulations, which was 400 ns (see Figure 1d). One structure for H1 and H3 each showed partial unfolding in the region adjacent to the fusion peptide, increasing the overall RMSD to ≃3 Å. Overall, these RMSD values are similar to the resolution of X-ray structures of related HAs [12,34,35], thus we expected that the designs, when expressed recombinantly, would be stable in a solution. As a final computational test before expression, we docked the three Fabs of the bnAb FI6V3 [12] from its X-ray structure in the complex with A/Aichi/2/1968(H3) hemagglutinin to each monomer in the headless H3 design. The docked Ab/antigen interfaces did not show steric clashes (Figure 1c). Starting from the docked complex, we simulated the Ab-bound trimer in 400 ns of MD simulations. Despite significant fluctuations of the Fabs, reaching RMSDs of ∼7.5 Å, all three interfaces remained stable in the simulation (Figure 1e,f), i.e., all Fabs remained bound to the HA trimer in the same binding pose. Thus, the computational tests suggested that the designed structure presents the correct epitope for binding to the FI6V3 bnAb. To establish an experimental proof of concept for the CXV-stabilized designs, we first expressed the H3 design in a small-scale (30 mL) 293F Human Embryonic Kidney (HEK) expression system from Genscript, and obtained a protein yield of 9.8 mg. A reduced SDS-PAGE showed a single well-defined band corresponding to the design (H3* in Figure 1g), and size-exclusion chromatography (SEC-HPLC) indicated that the eluate was primarily trimeric (H3-CXVH in Figure 1h). Encouraged by the positive result, we re-expressed the H3 design, this time along with H1 and H5 designs, with the SpyTag peptide sequence added at the C-terminus for conjugation to mi3 NPs [39] via the SpyCatcher protein [38]. The expression yielded 6.8 mg, 5.9 mg, and 8.9 mg for designs H3, H1, and H5, respectively. The expression of H3 was slightly lower than in the previous case, possibly because of a disordered C-terminal SpyTag. The three designs appeared trimeric by SEC-HPLC (Figure 1h). We also attempted the production of an H3 design in which the linkers optimized by Corbett et al. [29] for group II HAs were changed to the GSG repeats that Yassine et al. [35] used to produce headless H1. The experiment showed no significant expression, confirming that linker optimization and a strong trimerization domain are both necessary to produce a stable headless H3. Because our goal was to test the feasibility of CXV as an HA trimerization domain, rather than optimizing a vaccine immunogenicity profile, we used the human CXV sequence for our designs. However, because human proteins could potentially result in unwanted off-target immunogenicity [58], it may be desirable to use non-human sequences in future human vaccines. To evaluate the sensitivity of the design to the choice of the CXV homolog, we re-expressed the headless design H1 using as the trimerization domain (i) CXV from zebrafish (GenBank Acc. No. Q05H57) and (ii) the 52-residue N-terminal trimerization domain of bacteriophage-associated Hyaluronidase (GenBank Acc. No. Q9A0M7) [59], a homolog of CXV [42]. The sequence similarity between the human CXV domain (GenBank Acc. No. P39059) and the zebrafish and bacteriophage domains was 44% and 40%, respectively, using the blosum62 substitution matrix. With the zebrafish domain, the expression level was somewhat lower than that of the human CXV (4.4 mg vs. 5.9 mg), but the design remained trimeric by SEC-HPLC (Figure S2). The bacteriophage domain did not result in significant expression, although the protein was detectable in a Western blot. These results suggest that the use of non-human multiplexins or their homologs is possible, but further work is needed if bacterial homologs are desired. As a step towards producing headless HA antigens for any subtype, we selected eight additional influenza HAs of subtypes H2, H6, H7, H9, H10, H13, H16, and H17 (listed in Table S2), and designed and expressed corresponding headless antigens, as described above for H1, H3, and H5. In choosing the specific strains as the basis for the headless designs, we focused only on those for which X-ray structures are available in the PDB. This allowed us to check whether the sequence modifications introduced into the designs would not cause steric clashes with the rest of the HA. Further, if the structures of our designs are solved in the future, they can be compared directly with the parent PDB structure. The expression (SDS-PAGE) and characterization (SEC-HPLC) data for the additional designs are provided in the Supporting Materials. All of them expressed at high levels, ranging from 3.4 to 10.3 mg per 30mL of culture (Table S2), and were predominantly trimeric by SEC-HPLC. Designs of H2 and H6 showed an earlier peak corresponding to higher-order oligomers, indicative of partial aggregation (Figure S4), which can be removed by an additional purification step based on SEC to isolate the trimers. Overall, the expression results suggest the feasibility of creating homotypic and mosaic NPs of various compositions to investigate or optimize different vaccines.

**Table 1:** Mammalian expression of headless influenza antigens (see Section 2); yield of purified protein per 30 mL of expression volume. In the design names, ST denotes SpyTag, and the letter after CXV indicates the organism (human, zebrafish, or Staphylococcus). CXVS denotes Hyaluronidase, a structural homolog of CXV from a bacteriophage that infects *S. pyogenes* [59]. GSG indicates that the linkers have been reverted to GSG repeats. The complete amino acid sequences are given in Table S1. N/A indicates that the expression was too low to quantify yield reliably.

Design	Yield (mg)
*H3-CXVH*	9.8
*H3-CXVH-ST*	6.8
*H3-CXVH-GSG*	N/A
*H1-CXVH-ST*	5.9
*H1-CXVS-ST*	N/A
*H1-CXVZ-ST*	4.4
*H5-CXVH-ST*	8.9

## 4. Discussion

**Figure 2:**
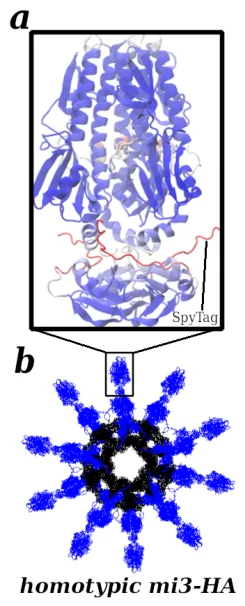
Mi3 nanoparticles (NPs) [39] can be conjugated to HA designs using the SpyTag system [38]; (**a**) headless HA antigen expressed with the SpyTag sequence; the design is colored by the predicted local distance difference (pLDD) metric [45]; (**b**) molecular model of the nanoparticles; the NP is in black, the HA designs are in blue.

**Figure 3:**
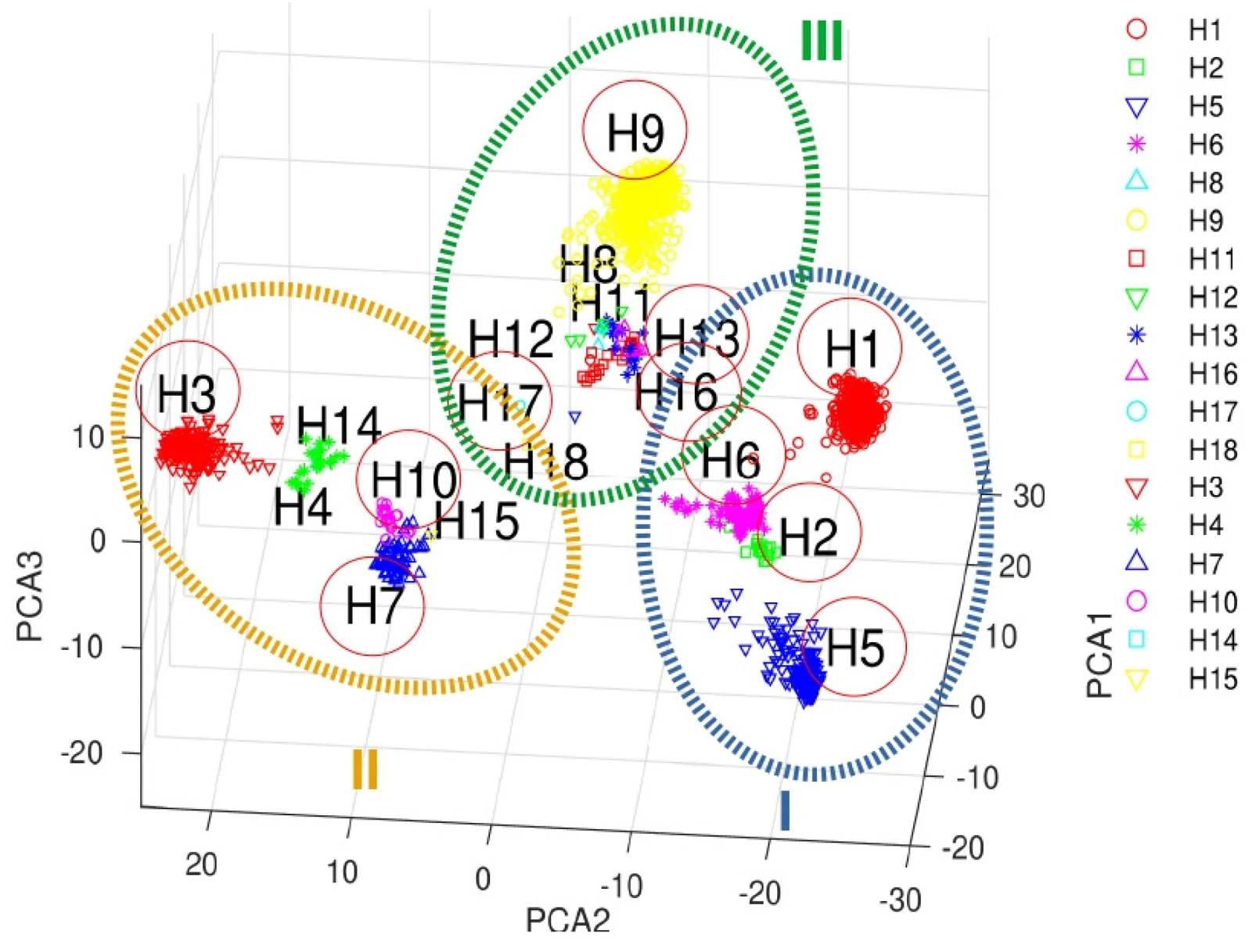
A possible future the axis labels in the figure are automatically drawn by MATLAB, and cannot be changed. study to optimize vaccine breadth. The scatter plot represents three-dimensional projections of HA sequences computed using principal component analysis (PCA). Red circles are drawn around subtypes that could be included in a hypothetical vaccine cocktail; the corresponding strains are listed with accession numbers in Table S2 and aligned in Table S3; expression data are provided in Figures S3 and S4. Large ovals are drawn around antigen subsets that could comprise a prime-boost-boost vaccine regimen.

The development of durable protective vaccines is hampered by the antigenic drift of the influenza genome, and by antigenic shifts, whereby segments of influenza genomes are reassorted between distant strains in a coinfected host, facilitating the emergence of pandemic strains. The elicitation of broadly neutralizing antibodies (bnAbs) that can neutralize a broad range of antigen variants, in contrast to strain-specific Abs that usually dominate the adaptive immune response, has been a focus of vaccine research. Because bnAbs against influenza tend to be rare and not well boosted in natural infections, their elicitation by immunization requires special considerations, such as the use of chimeric antigens [60], the masking of epitopes with glycans [17], or removing certain undesired epitopes [32]. In this study, we expanded on the last approach [29,35] to produce engineered groups I and II influenza hemagglutinin antigens in high yields. These antigens lack the immunodominant head (Figure 1b) and are stabilized in trimeric form using collagen XV proteins. Because the present antigens were expressed with a SpyTag [38], they can be conjugated to nanoparticles (NPs) for effective presentation to the immune system [25,27], (see Figure 2) without the need for genetic fusion. This approach makes it straightforward to create mosaic NPs of different HA trimers. A possible next step toward a universal flu vaccine is to optimize the breadth of NP vaccine responses, e.g., using immunizations with NPs of different compositions, as illustrated in Figure 3, which shows the landscape of influenza HA strains projected onto the three dominant principal components (see Section 2), and recently modeled computationally [55]. To this end, we selected eight additional influenza HAs of subtypes H2, H6, H7, H9, H10, H13, H16, and H17, for which X-ray structures are available (circled in Figure 3 and listed in Table S2), and designed and expressed the corresponding headless antigens. All designs were expressed at high levels (Table S2) and were predominantly trimeric by SEC-HPLC. To estimate the similarity between the strains chosen for the headless designs and recently circulating strains, we used the EpiFlu database (www.gisaid.org, accessed 25 February 2025) to download hemagglutinin sequences of strains submitted from 1 January 2025 to 1 July 2026. The chosen subtypes were H3 subclade K, H1(pdm09), H5, H7 and H9. For all subtypes except H7, the strains were isolated from human hosts. For H7, we did not find strains isolated from humans in the specified time period and selected avian hosts. We multiply aligned all strains within each subtype using MAFFT [61], and scored the alignment using the blosum62 matrix. The strains were ≥98% similar for subtypes H1,H3 and H5, and ≥90% similar for subtypes H7 and H9. Because of the high sequence similarity, we chose a strain randomly from each subtype set to compare with the strains used for the designs. The chosen strains were EPI5483321|A/Spain/HCB-000289/2026|EPI_ISL_20483432|H3N2, EPI5486947|A/South_Africa/PATH-CERI-C076790/2026|EPI_ISL_20484664|H1N1, EPI5181936|A/Michigan/90/2024|EPI_ISL_20374014|H5N1, EPI5281266|A/mallard_duck/Colorado/02060-001/2026|EPI_ISL_20406485|H7N2, and EPI5275080|A/Italy/1694/2026|EPI_ISL_20404890|H9N2, where the first word is the strain identifier in the EpiFlu database. A multiple sequence alignment between the above strains and the design strains was used to calculate the sequence identity and similarity (shown in Figures S5 and S6). The sequence identity between the corresponding subtypes was in the range 72–84%, and the sequence similarity using the blosum62 matrix was in the range 77–86%. An interesting future study would be to investigate whether a stalk-based influenza vaccine with such levels of similarity to circulating strains could significantly reduce or prevent symptomatic illness. To suggest possible antigen cocktails for such a vaccination study, large ovals are drawn around groups of adjacent HAs, which could be incorporated into different vaccination regimens (e.g., I/II/II for prime/boost/boost), for exploring vaccines with coarse-grained coverage of the influenza HA landscape. Evaluating cross-protection reliably would require viral challenges with several different influenza strains, and, therefore, a substantially larger cohort of mice than the one used here. Further, characterization of the vaccine-induced B cell repertoire would be needed to determine whether the vaccines elicit actual bnAbs, rather than broadly protective sera, which could be composed of many strain-specific antibodies [62]. A complementary preliminary study would be to evaluate the binding of the present antigens to a large panel of known bnAbs, which would shed light on the different epitopes presented by the antigens to the immune system.

The present study advances the antigen engineering of vaccines that display conserved influenza “headless” stalks to elicit broadly neutralizing antibodies. However, although HA stalk-based vaccines appear promising, it is unclear whether they could form the sole basis for universal vaccines. For example, anti-stalk bnAbs tend to have lower potency, compared to strain-specific Abs [18]. On the other hand, Abs can employ different mechanisms of protection, e.g., labeling infected cells for destruction by Ab-directed cellular cytotoxicity (ADCC), or the inhibition of membrane fusion after phagocytosis by the host cell, rather than the blockage of viral binding to cells, and comparisons between Abs that bind different epitopes are not always straightforward. Alternatively, HA stalk-based vaccines could supplement traditional vaccines in young patients to guide early immune imprinting towards enhancing the elicitation of anti-stalk bnAbs. We hope that our study will facilitate further development of headless flu vaccines.

## Data Availability

The information to reproduce the findings in this manuscript is contained within this manuscript and the Supplementary Materials.

## Supplementary Materials

The following supporting information can be downloaded at: https://www.mdpi.com/article/10.3390/antib15040064/s1.
