# Supplementary material for: Computational Design and Expression of Headless Influenza Hemagglutinin Antigens Toward a Modular Universal Nanoparticle Vaccine
Source: Antibodies (Basel). 2026 Jul 27;15(4):64. doi: 10.3390/antib15040064 (PMC13510027; doi:10.3390/antib15040064)
Supplement: Supplementary file 1 [file antibodies-15-00064-s001.zip › vomk26si.pdf]

Supporting Information

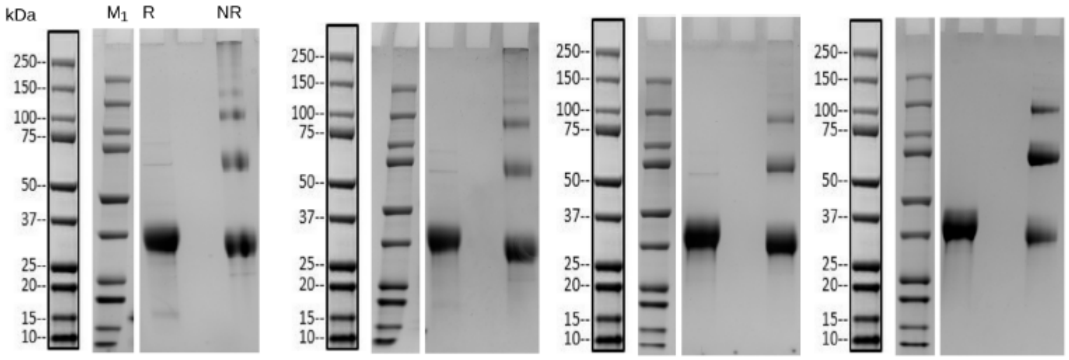

Figure S1. Characterization of headless designs. Left to right : H3-CXVH, H3-CXVH-ST, H1-CXV-ST, and H5-CXVH-ST. Reduced and non-reduced SDS-PAGE gels are shown in middle and fifth lanes, respectively. Although the non-reduced gels show multiple bands consistent with multiple oligomeric states, the HPLS-SEC chromatograms indicate that the designs are predominantly trimeric (Fig. 1h).

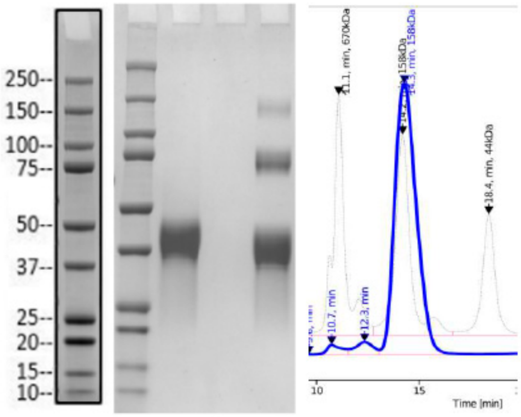

Figure S2. Characterization of headless design H1-CXVZ-ST, which has the trimerization domain from zebrafish. In the SDS-PAGE gel (left), the middle and fifth lanes correspond to reducing and non-reducing conditions, respectively.

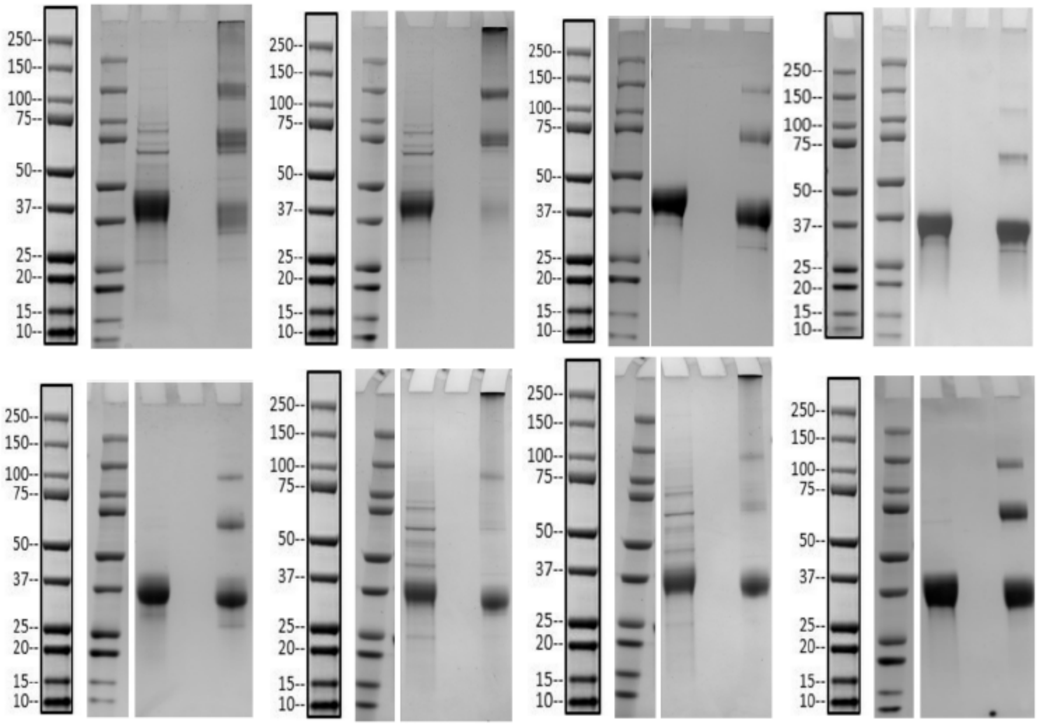

Figure S3. Reduced and non-reduced SDS-PAGE gels of headless designs. Left to right and top to bottom : H2, H6, H7, H9, H10, H13, H16, H17. SDS-PAGE for H1, H3, and H5 are shown in Fig. S1. The middle and fifth lanes of each gel correspond to reducing and non-reducing conditions, respectively. Although the non-reduced gels show multiple bands consistent with multiple oligomeric states, the HPLS-SEC chromatograms indicate that the designs are predominantly trimeric (Fig. S4).

Table S1. Multiple sequence alignment of influenza headless antigens based on subtypes H1, H3, and H5 in Table 1. The H1 and H3 sequences are modified from Yassine et al. [35] and Corbett et al. [29], respectively, by adding a collagen XV (cXV) trimerization domain, and a SpyTag.[38] The H5 is based on the strain A/DUCK/GUANGXI/2396/2004 (PDB: 4JUN). The top sequence is colored to indicate domains, as follows: signal peptide, linker, cXV, SpyTag, Histdine tag. In the sequence names, ST denotes SpyTag, GSG indicates that the linkers have been reverted to GSG repeats, and the letter after CXV indicates organism (Human, Zebrafish, or Staphylococcus).

H3-CXVH-ST	MGWSCIILFLVATATGVHSSTATLCLGHHAVPNGTIVKTITNDQIEV-TNATELVFPGCGV	60
H3-CXVH	MGWSCIILFLVATATGVHSSTATLCLGHHAVPNGTIVKTITNDQIEV-TNATELVFPGCGV	60
H3N2GS3	MGWSCIILFLVATATGVHSSTATLCLGHHAVPNGTLVKTITDDQIEV-TNATEL---GSG-	56
H1-CXVH-ST	MGWSCIILFLVATATGVHS-ADTICIGY-HANNSTDTVDTVLEKNVTVTHSVNL---GSG-	55
H1-CXVS-ST	MGWSCIILFLVATATGVHS--DTICIGY-HANNSTDTVDTVLEKNVTVTHSVNL---GSG-	54
H1-CXVZ-ST	MGWSCIILFLVATATGVHS--DTICIGY-HANNSTDTVDTVLEKNVTVTHSVNL---GSG-	54
H5-CXVH-ST	MGWSCIILFLVATATGVHSKSDQICIGYHANNSTEQVD-TIMEKNVTVTHAQDI---GSG-	56
	******************** :*:*:** . *.*: :.:: **:: :: *.*	
H3-CXVH-ST	LKLATGMRNVPELKQTRGIFGAIAGFIENGWEGMVDGWYGFRHQN-SEGIGQAADLKSTQA	120
H3-CXVH	LKLATGMRNVPELKQTRGIFGAIAGFIENGWEGMVDGWYGFRHQN-SEGIGQAADLKSTQA	120
H3-CXVH-GSG	LKLATGMRNVPE-KQTRGLFGAIAGFIENGWEGMIDGWYGFRHQN-SEGTGQAADLKSTQA	115
H1-CXVH-ST	LRMVTGLRNIPQ-RETRGLFGAIAGFIEGGWTGMVDGWYGY-HHQNEQGSGYAADQKSTQN	114

H1-CXVS-ST	LRMVTGLRNIPQ-RETRGLFGAIAGFIEGGWTGMVDGWYGY-HHQNEQGSGYAADQKSTQN	113
H1-CXVZ-ST	LRMVTGLRNIPQ-RETRGLFGAIAGFIEGGWTGMVDGWYGY-HHQNEQGSGYAADQKSTQN	113
H5-CXVH-ST	LVLATGLRNSPQ-RETRGLFGAIAGFIEGGWQGMVDGWYGY-HHSNEQGSGYAADKESTQK	115
	* :.**:** *: ::***:*********.** **:*****::*.*.:* * *** :***	
H3-CXVH-ST	AINQINGMVNRVIELMEQGGPDCYLAELLVALLNQHVIDLT-DSEMRKLFERTKKQLRENA	180
H3-CXVH	AINQINGMVNRVIELMEQGGPDCYLAELLVALLNQHVIDLT-DSEMRKLFERTKKQLRENA	180
H3-CXVH-GSG	AIDQINGKLNRVIEKTGSGGSGTYNAELLVALENQHTIDLT-DSEMNKLFEKTRRQLRENA	175
H1-CXVH-ST	AINGITNMVNSVIEKMGSGGSGTDLAELLVLLLNERTLDFHDSNVKN-LYEKVKSQLKNNA	174
H1-CXVS-ST	AINGITNMVNSVIEKMGSGGSGTDLAELLVLLLNERTLDFHDSNVKN-LYEKVKSQLKNNA	173
H1-CXVZ-ST	AINGITNMVNSVIEKMGSGGSGTDLAELLVLLLNERTLDFHDSNVKN-LYEKVKSQLKNNA	173
H5-CXVH-ST	AIDGVTNMVNSIIDKMGSGGSGTDLAELLVLMLNERTLDFHDSNVKN-LYDRVRLQLRDNA	175
	**: :.. :* :*: .**.. ***** : *:::.:*: **:::.:*:::: **::**	
H3-CXVH-ST	EDMGNGCFKIYHKCDNACIGSIRNGTYDHDVYRDEAL-NNRFQIKSGGNLVTAFSNMD-DM	239
H3-CXVH	EDMGNGCFKIYHKCDNACIGSIRNGTYDHDVYRDEAL-NNRFQIKSGGNLVTAFSNMD-DM	239
H3-CXVH-GSG	EEMGNGCFKIYHKCDNACIESIRNGTYDHDVYRDEAL-NNRFQIKPGSNLVTAFSNMD-DM	234
H1-CXVH-ST	KEIGNGCFEFYHKCNNECMESVKNGTYDYPKYSEESKLNRE-KIDPGSNLVTAFSNMD-DM	233
H1-CXVS-ST	KEIGNGCFEFYHKCNNECMESVKNGTYDYPKYSEESKLNRE-KIDPGSLRVQFKRMKAAEW	233
H1-CXVZ-ST	KEIGNGCFEFYHKCNNECMESVKNGTYDYPKYSEESKLNREKIDPG-SASSTLLRFVSVQL	233
H5-CXVH-ST	KELGNGCFEFYHKCDNECMESVRNGTYDYPQYSEEARLKREE-ISPGSNLVTAFSNMD-DM	234
	:::*****::****:* *: *::*****: * :*: :* :*..*. :	
H3-CXVH-ST	LQKAHLVIEGTFIYLRDSTEFFIRVRDGWKKLQLGELIPIP-AGSGAHIVMVDAYKPTGSG	299
H3-CXVH	LQKAHLVIEGTFIYLRDSTEFFIRVRDGWKKLQLGELIPIPA------------------	281
H3-CXVH-GSG	LQKAHLVIEGTFIYLRDSTEFFIRVRDGWKKLQLGELIPIPA------------------	276
H1-CXVH-ST	LQKAHLVIEGTFIYLRDSTEFFIRVRDGWKKLQLGELIPIP-AGSGAHIVMVDAYKPTGSG	293
H1-CXVS-ST	ARSDVILLESEIGFETDTGFARAGDGHN-RFSDLGYI--SPLGSGAHIVMVDAYKPTGSG	290
H1-CXVZ-ST	MQQQVFVQDGSLAFITDSSK-LYLKVPRGWREVQLGVLLESYPGSGAHIVMVDAYKPTGSG	293
H5-CXVH-ST	LQKAHLVIEGTFIYLRDSTEFFIRVRDGWKKLQLGELIPIP-AGSGAHIVMVDAYKPTGSG	294
	:. :: :. : : *: . : :** :	
H3-CXVH-ST	HHHHHH	305
H3-CXVH	HHHHHH	287
H3-CXVH-GSG	HHHHHH	282
H1-CXVH-ST	HHHHHH	299
H1-CXVS-ST	HHHHHH	296
H1-CXVZ-ST	HHHHHH	299
H5-CXVH-ST	HHHHHH	300
	******	

Table S2. Influenza strains suggested for a hypothetical broadly-neutralizing vaccine. The sequences of designed antigens are aligned in Table S3, and expression data are given in Fig. S3. The yield of designed antigens is given in milligrams of protein per 30mL of expression culture.

ORIGINAL STRAIN NAME	ACCESSION #, PDB ID	YIELD OF DESIGN(MG)
A/Kagoshima/1/2009(H1N1)	ACT22035.1, 3LZG	5.9
A/Japan/305/1957(H2N2)	ACV49600, 4HLZ	4.1
A/Aichi/2/1968(H3)	AAA43178.1, 1EO8	6.8
A/duck/Guangxi/2396/2004(H5N1)	ABC66534, 4JUN	8.9
A/chicken/Guangdong/S1312/2010(H6N2)	AHJ58072, 5BNY	4.0
A/Hangzhou/1/2013(H7N9)	AGI60301, 4BSA	10.1
A/Swine/Hong_Kong/9/98(H9)	EPI6943, 1JSD	5.4
A/chicken/Germany/N/1949(H10N7)	ABI84534, 4D00	10.3
A/gull/Maryland/704/1977(H13)	BAA14338.1, 4KPQ	3.4
A/black-headed_gull/Sweden/2/99(H16)	AAV91214.1, 4F23	6.5
A/little yellow-shouldered bat/Guatemala/060/2010(H17)	AFC35438.1, 4H32	7.5

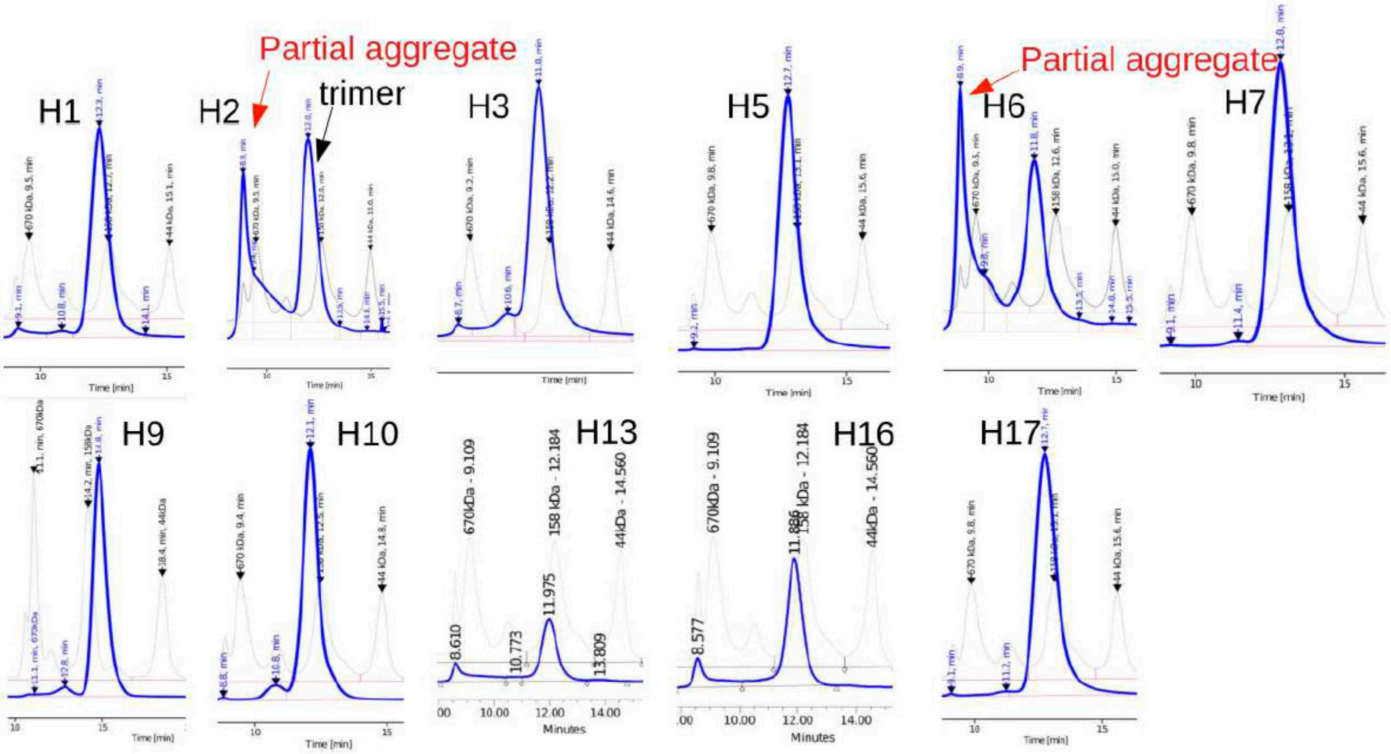

Figure S4. SEC-HPLC chromatograms of headless designs. All designs are expressed with a cXV human trimerization domain and a SpyTag. Designs H2 and H6 show higher-order oligomers suggestive of aggregation, and therefore would require an additional purification step to separate the trimers.

Table S3. Multiple sequence alignment of influenza headless antigens. The H1, H3 and H5 sequences are the same as those in Table S1. Strain names and PDB IDs of corresponding full-length HAs are given in Table S2. The top sequence is colored to indicate domains, as in Table S1: signal peptide, linker, cXV, SpyTag, Histdine tag. In the sequence names, CXVH indicates human multiplexin, and ST denotes SpyTag.

H1-CXVH-ST	MGWSCIILFLVATATGVHS-ADTICIGY-HANNSTDTVDTVLEKNVTVTHSVNL---GSG-	55
H2-CXVH-ST	MGWSCIILFLVATATGVHSRGDQICIGYHANNSTEKVD-TILERNVTVTHAKDI---GSG-	56

H3-CXVH-ST	MGWSCIILFLVATATGVHSSTATLCLGHHAVPNGTIVKTITNDQIEV-TNATELVFPGCGV	60
H5-CXVH-ST	MGWSCIILFLVATATGVHSKSDQICIGYHANNSTEQVD-TIMEKNVTVTHAQDI---GSG-	56
H6-CXVH-ST	MGWSCIILFLVATATGVHSKSDKICIGYHANNSTTKVD-TILEKNVTVTHSVEL---GSG-	56
H7-CXVH-ST	MGWSCIILFLVATATGVHSNADKICLGHHAVSN-GTKVNTLTERGVEVVNATELVFPGCGV	60
H9-CXVH-ST	MGWSCIILFLVATATGVHS--DKICIGHQSTNSTETVD-TLTETNVPVTHAKELVFPGCGV	58
H10-CXVH-ST	MGWSCIILFLVATATGVHSGLDKICLGHHAVANGTIVKTLTNEQEEV-TNATELVFPGCGV	60
H13-CXVH-ST	MGWSCIILFLVATATGVHSHADRICVGYLSTNSSERVDTLLENGVPVTSSI-DLVFPGCGV	60
H16-CXVH-ST	MGWSCIILFLVATATGVHSRADKICIGYLSNNSTDTVDTLTENGVPVTSSI-DLVFPGCGV	60
H17-CXVH-ST	MGWSCIILFLVATATGVHSLG-DRICIGYQANQNNQTVNTLLEQNVPVTGAQEI---GSG-	56
	******************* :*:*: : . *.*: : *. : :: *.*	
H1-CXVH-ST	LRMVTGLRNIPQR-ETRGLFGAIAGFIEGGWTGMVDGWYGY-HHQNEQGSGYAADQKSTQN	114
H2-CXVH-ST	LVLATGLRNVPQI-ESRGLFGAIAGFIEGGWQGMVDGWYGYHHSND-QGSGYAADKESTQK	115
H3-CXVH-ST	LKLATGMRNVPELKQTRGIFGAIAGFIENGWEGMVDGWYGFRHQN-SEGIGQAADLKSTQA	120
H5-CXVH-ST	LVLATGLRNSPQR-ETRGLFGAIAGFIEGGWQGMVDGWYGY-HHSNEQGSGYAADKESTQK	115
H6-CXVH-ST	LRLATGLRNVPQI-ETRGLFGAIAGFIEGGWTGMIDGWYGYHHENS-QGSGYAADKESTQK	115
H7-CXVH-ST	LKLATGMKNVPEIPKGRGLFGAIAGFIENGWEGLIDGWYGFRHQNAQGEG-TAADYKSTQS	120
H9-CXVH-ST	LKLAVGLRNVPAR-SSRGLFGAIAGFIEGGWPGLVAGWYGFQWSND-QGVGMAADRDSTQK	117
H10-CXVH-ST	LMLATGMRNVPELIQGRGLFGAIAGFLENGWEGMVDG-WYGFRHQNAQGTGQAADYKSTQA	120
H13-CXVH-ST	LKLATGLRNVPAI-SNRGLFGAIAGFIEGGWPGLINGWYGFQHQNEQGT-GIAADKESTQK	119
H16-CXVH-ST	LKLATGLRNVPSI-VERGLFGAIAGFIEGGWPGLINGWYGFQHQNEQGT-GIAADKTSTQK	119
H17-CXVH-ST	LMLATGLRNNPQM-EGRGLFGAIAGFIEGGWQGMIDGWYGY-HHENQEGSGYAADKEATQK	115
	* :..*::* * **:*******:*.** *:: ****:: .* :* * *** :**	
H1-CXVH-ST	AINGITNMVNSVIEKMGSGGSGTDLAELLVLLLNERTLDFHDSNVKN-LYEKVKSQLKNNA	174
H2-CXVH-ST	AFDGITNKVNSVIEKMGSGGSGTDLAELLVLMLNERTLDFHDSNVKN-LYDKVRMQLRDNV	175
H3-CXVH-ST	AINQINGMVNRVIELMEQGGPDCYLAELLVALLNQHVIDLT-DSEMRKLFERTKKQLRENA	180
H5-CXVH-ST	AIDGVTNMVNSIIDKMGSGGSGTDLAELLVLMLNERTLDFHDSNVKN-LYDRVRLQLRDNA	175
H6-CXVH-ST	AIDGITNKVNSIIDKMGSGGSGTDLAEL-LVLLLNERTLDLHDANVKNLHEKVRSQLRDNA	175
H7-CXVH-ST	AIDQITGMVNRVIELMEQGGPDCYLAELLVAMLNQHVID-LADSEMDKLYERVKRQLRENA	180
H9-CXVH-ST	AIDKITSIVNNIVDKMEQGGPDCYNAELLVLLINQKTLDEHDANVNNLYN-KVKRALGSNA	177
H10-CXVH-ST	AIDQITGMVNRVVELMEQGGPDCYLAELLVAMLNQHVIDMADSEMRNLY-ERVRKQLRQNA	180
H13-CXVH-ST	AIDQITTKINNIIDKMEQGGPDCYLAELLVL-LENDKTLDMHDANVKNLHEQVRRELKDNA	179
H16-CXVH-ST	AINEITTKINNIIEKMEQGGPDCYLAEL-LVLIENDRTLDLHDANVRNLHEQIKRALKDNA	179
H17-CXVH-ST	AVDAITNMVNSIIDKMGSGGSGSDLTELLVLLLNERTLDFHDANVKNLFEKV-KAQLKDNA	175
	.: :. : ::: * .**.. :**** : *:..:* *::: :*.:: : * .*.	
H1-CXVH-ST	KEIGNGCFEFYHKCNNECMESVKNGTYDYPKYSEESKLNREKID-PGSNLVTAFSNMDDM	233
H2-CXVH-ST	KELGNGCFEFYHKCDDECMNSVKNGTYDYPKYEEESKLNRNEIK-PGSNLVTAFSNMDDM	234
H3-CXVH-ST	EDMGNGCFKIYHKCDNACIGSIRNGTYDHDVYRDEALNNRFQIK-SGGNLVTAFSNMDDM	239

H5-CXVH-ST	KELGNGCFEFYHKCDNECMESVRNGTYDYPQYSEEARLKREEIS-PGSNLVTAFSNMDDM	234
H6-CXVH-ST	NDLGNGCFEFWHKCNNECMESVKNGTYDYPKYQKESRLNRQKIE-PGSNLVTAFSNMDDM	234
H7-CXVH-ST	EEDGTGCFEIFHKCDDDCMASIRNNTYDHSKYREEAMQNRIQID-SGGNLVTAFSNMDDM	239
H9-CXVH-ST	MEDGKGCFELYHKCDDQCMETIRNGTYNRR-KYREESRLERQKIEGSGSNLVTAFSNMDDM	237
H10-CXVH-ST	EEDGKGCFEIYHACDDSCMESIRNNTYDHSQYREEALLNRLNIN-SGGNLVTAFSNMDDM	239
H13-CXVH-ST	IDEGNGCFELLHKCNDSCMETIRNGTYDHTEYAEESKLKRQEID-PGSNLVTAFSNMDDM	238
H16-CXVH-ST	IDEGDGCFSILHKCNDSCMETIRNGTYNHEDYKEESQLKRQEIE-PGSNLVTAFSNMDDM	238
H17-CXVH-ST	IDEGNGCFLLLHKCNNSCMDDIKNGTYKYMDYREESHIEKQKID-PGSNLVTAFSNMDDM	234
	: * *** : * *:: *: ::*.**. * .*: :: :*. .*.************	
H1-CXVH-ST	LQKAHLVIEGTFIYLRDSTEFFIRVRDGWKKLQLGELIPIP-AGSGAHIVMVDAYKPTGSGHHHHHH	299
H2-CXVH-ST	LQKAHLVIEGTFIYLRDSTEFFIRVRDGWKKLQLGELIPIP-AGSGAHIVMVDAYKPTGSGHHHHHH	300
H3-CXVH-ST	LQKAHLVIEGTFIYLRDSTEFFIRVRDGWKKLQLGELIPIP-AGSGAHIVMVDAYKPTGSGHHHHHH	305
H5-CXVH-ST	LQKAHLVIEGTFIYLRDSTEFFIRVRDGWKKLQLGELIPIP-AGSGAHIVMVDAYKPTGSGHHHHHH	300
H6-CXVH-ST	LQKAHLVIEGTFIYLRDSTEFFIRVRDGWKKLQLGELIPIP-AGSGAHIVMVDAYKPTGSGHHHHHH	300
H7-CXVH-ST	LQKAHLVIEGTFIYLRDSTEFFIRVRDGWKKLQLGELIPIP-AGSGAHIVMVDAYKPTGSGHHHHHH	305
H9-CXVH-ST	LQKAHLVIEGTFIYLRDSTEFFIRVRDGWKKLQLGELIPIP-AGSGAHIVMVDAYKPTGS-HHHHHH	302
H10-CXVH-ST	LQKAHLVIEGTFIYLRDSTEFFIRVRDGWKKLQLGELIPIP-AGSGAHIVMVDAYKPTGSGHHHHHH	305
H13-CXVH-ST	LQKAHLVIEGTFIYLRDSTEFFIRVRDGWKKLQLGELIPIP-AGSGAHIVMVDAYKPTGSGHHHHHH	304
H16-CXVH-ST	LQKAHLVIEGTFIYLRDSTEFFIRVRDGWKKLQLGELIPIP-AGSGAHIVMVDAYKPTGSGHHHHHH	304
H17-CXVH-ST	LQKAHLVIEGTFIYLRDSTEFFIRVRDGWKKLQLGELIPIP-AGSGAHIVMVDAYKPTGSGHHHHHH	300
	** ******	

	EPI5486947|H1|	EPI5483321|H3|	EPI5181936|H5|	EPI5281266|H7|	EPI5275080|H9|	H1-stalk	H2-stalk	H3-stalk	H5-stalk	H6-stalk	H7-stalk	H9-stalk	H10-stalk	H13-stalk	H16-stalk	H17-stalk
EPI5486947|H1|	100															
EPI5483321|H3|	42.57	100														
EPI5181936|H5|	62.83	40.56	100													
EPI5281266|H7|	41.26	49.82	43.15	100												
EPI5275080|H9|	50.37	39.3	50	40.59	100											
H1-stalk	71.86	46.73	64.82	49.25	54.27	100										
H2-stalk	67.5	47	70	50.5	57.5	79.9	100									
H3-stalk	46.83	80.49	44.39	61.46	42.44	52.26	49.5	100								
H5-stalk	63.5	46.5	79.5	51	55.5	80.4	86	50.5	100							
H6-stalk	64.5	48.5	66	52.5	56.5	82.41	81.5	51.5	81.5	100						
H7-stalk	48.29	60.49	46.34	76.59	49.76	54.27	54.5	74.15	53	53.5	100					
H9-stalk	55.17	45.81	55.67	52.71	83.74	58.79	60.5	52.22	60	61	59.61	100				
H10-stalk	50.24	61.95	48.78	69.27	50.73	53.77	55	77.56	55.5	53	80.98	59.61	100			
H13-stalk	54.41	46.08	56.37	53.43	64.22	60.3	61	51.96	61	65.5	58.82	70.94	57.84	100		
H16-stalk	51.96	45.59	52.45	50.98	61.76	60.3	58.5	53.43	58	63.5	58.82	69.95	56.86	84.8	100	
H17-stalk	56	45.5	55	46.5	52	69.35	69	47.5	69	73	50	57	48.5	62	59.5	100

Figure S5. Sequence identity between the antigens used for the headless designs (H1-stalk to H17-stalk) and recently circulating influenza strains (see main text). The recent strains are

EPI5483321|A/Spain/HCB-000289/2026|EPI_ISL_20483432|H3N2,
EPI5486947|A/South_Africa/PATH-CERI-C076790/2026|EPI_ISL_20484664|H1N1,
EPI5181936|A/Michigan/90/2024|EPI_ISL_20374014|H5N1,.

EPI5281266 | A/mallard_duck/Colorado/02060-001/2026 | EPI_ISL_20406485 | H7N2,and EPI5275080 | A/Italy/1694/2026 | EPI_ISL_20404890 | H9N2, where the first word is the strain identifier in the EpiFlu database (www.gisaid.org). The sequences were multiply aligned using MAFFT.[61]

<table>
<tr><th></th><th>EPI5486947|H1|</th><th>EPI5483321|H3|</th><th>EPI5181936|H5|</th><th>EPI5281266|H7|</th><th>EPI5275080|H9|</th><th>H1-stalk</th><th>H2-stalk</th><th>H3-stalk</th><th>H5-stalk</th><th>H6-stalk</th><th>H7-stalk</th><th>H9-stalk</th><th>H10-stalk</th><th>H13-stalk</th><th>H16-stalk</th><th>H17-stalk</th></tr>
<tr><td>EPI5486947|H1|</td><td>100</td><td></td><td></td><td></td><td></td><td></td><td></td><td></td><td></td><td></td><td></td><td></td><td></td><td></td><td></td><td></td></tr>
<tr><td>EPI5483321|H3|</td><td>53.53</td><td>100</td><td></td><td></td><td></td><td></td><td></td><td></td><td></td><td></td><td></td><td></td><td></td><td></td><td></td><td></td></tr>
<tr><td>EPI5181936|H5|</td><td>72.12</td><td>51.11</td><td>100</td><td></td><td></td><td></td><td></td><td></td><td></td><td></td><td></td><td></td><td></td><td></td><td></td><td></td></tr>
<tr><td>EPI5281266|H7|</td><td>52.79</td><td>59.23</td><td>53.33</td><td>100</td><td></td><td></td><td></td><td></td><td></td><td></td><td></td><td></td><td></td><td></td><td></td><td></td></tr>
<tr><td>EPI5275080|H9|</td><td>59.11</td><td>50.92</td><td>59.07</td><td>51.11</td><td>100</td><td></td><td></td><td></td><td></td><td></td><td></td><td></td><td></td><td></td><td></td><td></td></tr>
<tr><td>H1-stalk</td><td>77.39</td><td>57.29</td><td>70.85</td><td>59.3</td><td>63.32</td><td>100</td><td></td><td></td><td></td><td></td><td></td><td></td><td></td><td></td><td></td><td></td></tr>
<tr><td>H2-stalk</td><td>72.5</td><td>56</td><td>74.5</td><td>61</td><td>63</td><td>85.43</td><td>100</td><td></td><td></td><td></td><td></td><td></td><td></td><td></td><td></td><td></td></tr>
<tr><td>H3-stalk</td><td>57.07</td><td>82.93</td><td>53.17</td><td>67.8</td><td>53.66</td><td>62.81</td><td>60.5</td><td>100</td><td></td><td></td><td></td><td></td><td></td><td></td><td></td><td></td></tr>
<tr><td>H5-stalk</td><td>72.5</td><td>57.5</td><td>81</td><td>60.5</td><td>62.5</td><td>86.93</td><td>91</td><td>62.5</td><td>100</td><td></td><td></td><td></td><td></td><td></td><td></td><td></td></tr>
<tr><td>H6-stalk</td><td>72.5</td><td>58</td><td>69.5</td><td>61.5</td><td>64</td><td>88.44</td><td>88</td><td>62</td><td>86</td><td>100</td><td></td><td></td><td></td><td></td><td></td><td></td></tr>
<tr><td>H7-stalk</td><td>57.07</td><td>68.29</td><td>55.12</td><td>80.49</td><td>58.05</td><td>62.81</td><td>63.5</td><td>80.49</td><td>63</td><td>63.5</td><td>100</td><td></td><td></td><td></td><td></td><td></td></tr>
<tr><td>H9-stalk</td><td>64.53</td><td>55.17</td><td>62.07</td><td>61.58</td><td>86.21</td><td>67.34</td><td>69</td><td>63.55</td><td>68</td><td>69</td><td>67</td><td>100</td><td></td><td></td><td></td><td></td></tr>
<tr><td>H10-stalk</td><td>59.02</td><td>69.27</td><td>56.1</td><td>74.15</td><td>57.07</td><td>62.81</td><td>63.5</td><td>82.93</td><td>64.5</td><td>62.5</td><td>85.85</td><td>66.5</td><td>100</td><td></td><td></td><td></td></tr>
<tr><td>H13-stalk</td><td>65.2</td><td>54.9</td><td>63.73</td><td>60.78</td><td>71.08</td><td>70.35</td><td>72</td><td>61.76</td><td>70</td><td>75.5</td><td>67.65</td><td>79.31</td><td>67.16</td><td>100</td><td></td><td></td></tr>
<tr><td>H16-stalk</td><td>63.24</td><td>54.41</td><td>60.78</td><td>59.31</td><td>70.1</td><td>69.35</td><td>68.5</td><td>62.75</td><td>67.5</td><td>72.5</td><td>66.18</td><td>78.33</td><td>65.2</td><td>91.67</td><td>100</td><td></td></tr>
<tr><td>H17-stalk</td><td>65.5</td><td>55.5</td><td>64</td><td>58</td><td>60.5</td><td>76.88</td><td>77.5</td><td>59</td><td>78.5</td><td>80</td><td>60</td><td>67</td><td>62</td><td>71</td><td>70</td><td>100</td></tr>
</table>

Figure S6. See the caption to Fig. S5. Here, sequence similarity (not identity) was computed using the blosum62 scoring matrix.
