# Supplementary material for: Computational Design and Expression of Headless Influenza Hemagglutinin Antigens Toward a Modular Universal Nanoparticle Vaccine
Source: Antibodies (Basel). 2026 Jul 27;15(4):64. doi: 10.3390/antib15040064 (PMC13510027; doi:10.3390/antib15040064)
Supplement: Supplementary file 1 [file antibodies-15-00064-s001.zip › gels_hplc.zip!/H10-C15ST_Protein_COA.pdf]

Certificate of Analysis

**Name:**	H10VRCC15ST
**Type:**	Immune Recombinant Protein
**Shipping Condition:**	Dry Ice
**Lot Number:**	U760JXPQG0-4/P7IH001
**Expression Host :**	Mammalian
**Purification:**	AmMagTM Ni Magnetic Beads

**Total Amount:**	10.30 mg. 1.00 ml/tube, 10 tubes
**Concentration:**	1.03 mg/ml(by A280)
**Purity:**	≥65%(SDS-PAGE under non-reducing condition)
**Endotoxin:**	≤0.1 EU/mg
**Storage And Handling:**	Store at -80°C. Avoid repeated freezing and thawing
**Storage Buffer:**	PBS, pH 7.2

SDS-PAGE:

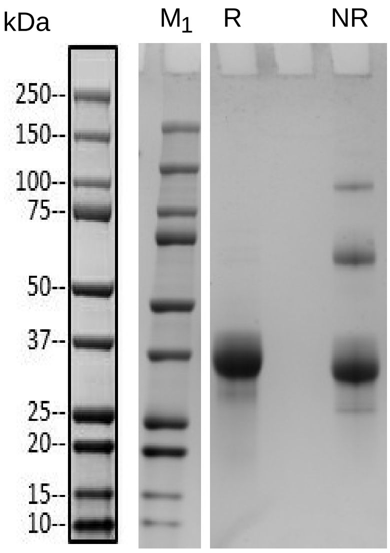

Lane M1: Protein Marker, Bio-rad, Cat. No. 1610374S, refer to annotated key on the left for size
R: Reducing condition
NR: Non-reducing condition

SEC-HPLC

Sample name: U760JXPQG0-1_After desalting

Instrument: GR16010687 Agilent 1260 **Location:** P1-A4 **Injection volume:** 19.0

Column Name: TSKgel G3000SWxl **Column No:** 197#

Mobile Phase: 0.1 mol/L Na2SO4 in 0.118 mol/L Phosphate Buffer(pH 6.7±0.3)

—— Sample
.... Standard

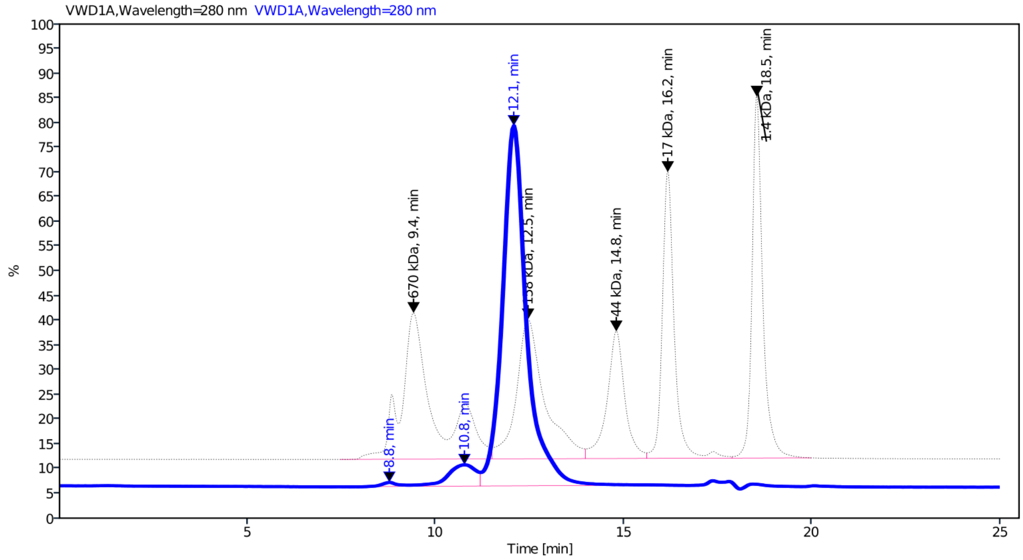

Signal: VWD1A,Wavelength=280 nm

RT [min]	Height	Width [min]	Area	Area%
8.8	0.4	1.2	10.8	1
10.8	1.8	1.9	96.6	6
12.1	31.2	3.8	1398.0	93
		Sum	1505.38	

GenScript USA, Inc.
860 Centennial Ave. Piscataway, NJ 08854
Tel: 1-732-885-9188

Appendix:

U760JXPQG0-1_H10VRCC15ST

Protein sequence: 305aa

MGWSCIILFLVATATGVHSGLDKICLGHHAVANGTIVKTLTNEQEEVTNATELVFPGCGVLMLATGMRNVPELIQGRGLFGAIAGFLENGWE
GMVDGWYGFRHQNAQGTGQAADYKSTQAAIDQITGMVNRVVELMEQGGPDCYLAELLVAMLNQHVIDMADSEMRNLYERVRKQLRQNA
EEDGKGCFEIYHACDDSCMESIRNNTYDHSQYREEALLNRLNINSGGNLVTAFSNMDDMLQKAHLVIEGTFIYLRDSTEFFIRVRDGWKKLQ
LGELIPIPAGSGAHIVMVDAYKPTGSGHHHHHH*

Certified by: Yinjia Zhu **Date:** 08/01/2023
