# Supplementary material for: Computational Design and Expression of Headless Influenza Hemagglutinin Antigens Toward a Modular Universal Nanoparticle Vaccine
Source: Antibodies (Basel). 2026 Jul 27;15(4):64. doi: 10.3390/antib15040064 (PMC13510027; doi:10.3390/antib15040064)
Supplement: Supplementary file 1 [file antibodies-15-00064-s001.zip › gels_hplc.zip!/H13-V3C15ST_Protein_COA.pdf]

GenScript
Make Research Easy

Certificate of Analysis

**Name:**	H134KPQV3C15ST
**Type:**	Recombinant protein
**Shipping Condition:**	Dry Ice
**Lot Number:**	U5539FALG0-14/P9IK001
**Expression Host :**	Mammalian
**Purification:**	Ni-Charged MagBeads

**Total Amount:**	3.44 mg. 1.00 ml/tube, 8 tubes
**Concentration:**	0.43 mg/ml(by A280)
**Purity:**	≥65%(SDS-PAGE under non-reducing condition) ;N/A(SEC-HPLC)
**Endotoxin:**	≤0.2 EU/mg
**Storage And Handling:**	Store at -80.0°C. Avoid repeated freezing and thawing
**Storage Buffer:**	PBS, pH 7.2

GenScript USA, Inc.
860 Centennial Ave. Piscataway, NJ 08854
Tel: 1-732-885-9188

SDS-PAGE:

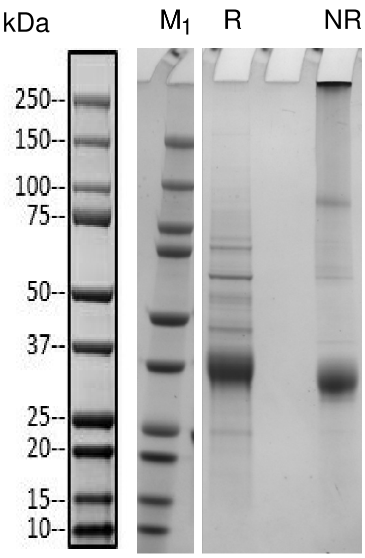

Lane M1: Protein Marker, Bio-rad, Cat. No. 1610374S, refer to annotated key on the left for size
R: Reducing condition
NR: Non-reducing condition

GenScript USA, Inc.
860 Centennial Ave. Piscataway, NJ 08854
Tel: 1-732-885-9188

SEC-HPLC

Sample Name: U5539FALG0-11_After desalting

Instrument: Arc PREMIER | Location: 1:A,8 | Sample Set: 10242023 PM

Column Name: TSKGel G3000SWxl | Column No: 27# | Injection Volume: 48.00 uL

Mobile Phase: 0.1mol/L Na2SO4 in 0.1mol/L Phosphate Buffer (pH 6.7±0.3)

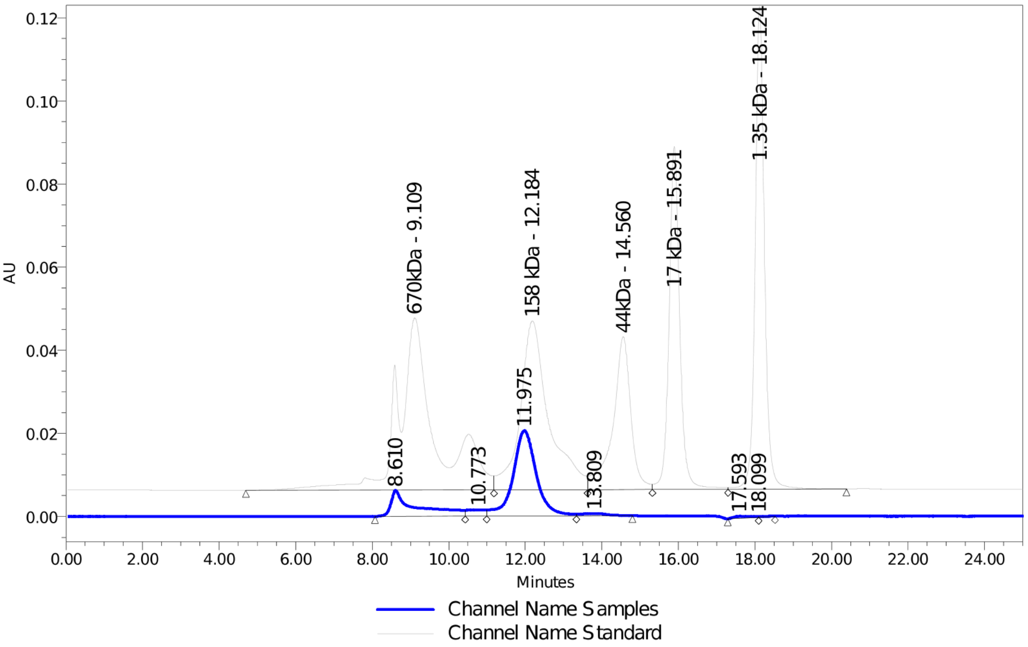

	Retention Time (min)	Width (sec)	Height (µV)	Area (µV*sec)	AreaPercentage
1	8.610	141.600	6236	284805	23
2	10.773	33.600	1516	49831	4
3	11.975	140.400	20545	868693	70
4	13.809	88.200	572	26658	2
5	17.593	48.300	469	13650	1
6	18.099	26.000	217	3834	0

GenScript USA, Inc.
860 Centennial Ave. Piscataway, NJ 08854
Tel: 1-732-885-9188

Appendix:

U5539FALG0-11_H134KPQV3C15ST

Full length protein chain:

Protein chain sequence: 304aa

MGWSCIILFLVATATGVHSHADRICVGYLSTNSSERVDTLLENGVPVTSSIDLVFPGCGVLKLATGLRNVPAISNRGLFGAIAGFIEGGWPGLINGWYGF
QHQNEQGTGIAADKESTQKAIDQITTKINNIIDKMEQGGPDCYLAELLVLLENDKTLDMHDANVKNLHEQVRRELKDNAIDEGNGCFELLHKCNDSCME
TIRNGTYDHTEYAEESKLKRQEIDPGSNLVTAFSNMDDMLQKAHLVIEGTFIYLRDSTEFFIRVRDGWKKLQLGELIPIPAGSGAHIVMVDAYKPTGSGH
HHHHH

Certified by: Zhangling Wang **Date:** 11/14/2023
