# Supplementary material for: Computational Design and Expression of Headless Influenza Hemagglutinin Antigens Toward a Modular Universal Nanoparticle Vaccine
Source: Antibodies (Basel). 2026 Jul 27;15(4):64. doi: 10.3390/antib15040064 (PMC13510027; doi:10.3390/antib15040064)
Supplement: Supplementary file 1 [file antibodies-15-00064-s001.zip › gels_hplc.zip!/H1-C15ST_Protein_COA.pdf]

Certificate of Analysis

**Name:**	H1VRCC15ST
**Type:**	Recombinant protein of immune products
**Shipping Condition:**	Dry Ice
**Lot Number:**	U4592IC100-9/P7IC001
**Expression Host :**	Mammalian
**Purification:**	HisTrap™ FF Crude

**Total Amount:**	5.85 mg. 3.00 ml/tube, 5 tubes
**Concentration:**	0.39 mg/ml(by A280)
**Purity:**	≥75%(SDS-PAGE under non-reducing condition)
**Endotoxin:**	≤0.6 EU/mg
**Storage And Handling:**	Store at -80°C.Avoid repeated freezing and thawing
**Storage Buffer:**	PBS, pH 7.2

GenScript USA, Inc.
860 Centennial Ave. Piscataway, NJ 08854
Tel: 1-732-885-9188

SDS-PAGE:

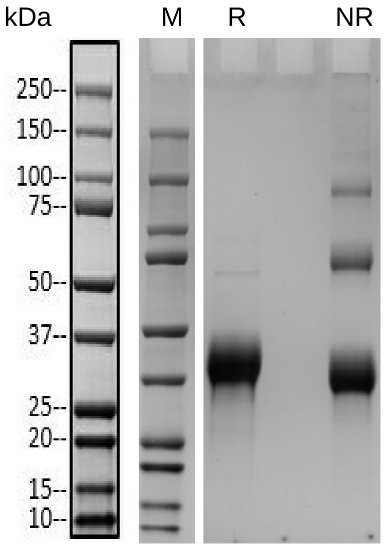

Lane M: Protein Marker, Bio-rad, Cat. No. 1610374S, refer to annotated key on the left for size
R: Reducing condition
NR: Non-reducing condition

GenScript
Make Research Easy

SEC-HPLC

Sample name: U4592IC100-6_after desalting

Instrument: GR16010687 **Location:** P1-B4 **Injection volume:** 53.0

Column Name: TSKgel G3000SWxl **Column No:** 172#

Mobile Phase: 0.1 mol/L Na2SO4 in 0.1 mol/L Phosphate Buffer(pH 6.7±0.3)

—— Sample
.... Standard

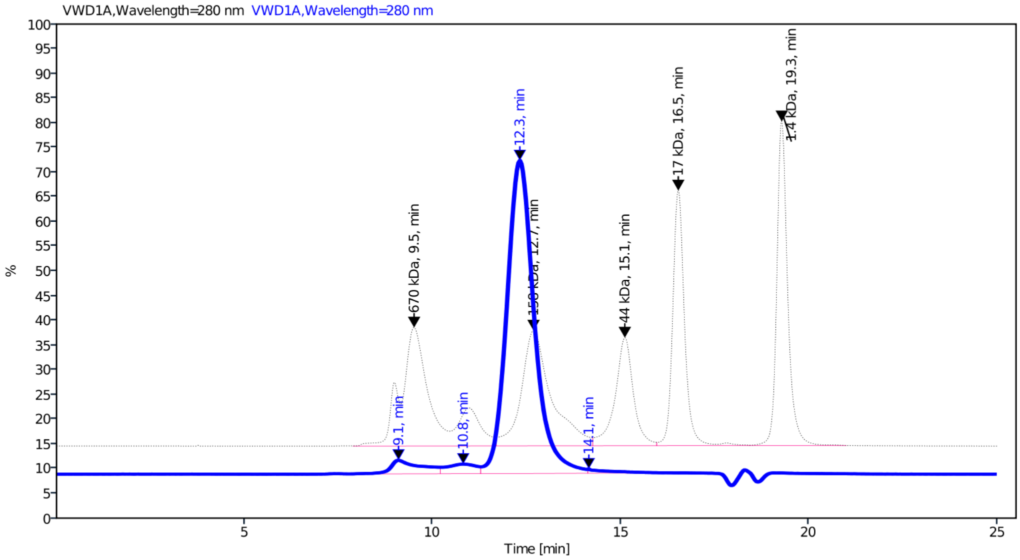

Signal: VWD1A,Wavelength=280 nm

RT [min]	Height	Width [min]	Area	Area%
9.1	1.4	2.4	79.1	5
10.8	1.0	1.1	52.5	3
12.3	31.6	2.8	1603.8	91
14.1	0.4	2.3	18.0	1
		Sum	1753.43	

GenScript USA, Inc.
860 Centennial Ave. Piscataway, NJ 08854
Tel: 1-732-885-9188

Appendix:

U4592IC100-6_H1VRCC15ST

Protein sequence: 299aa

MGWSCIILFLVATATGVHSADTICIGYHANNSTDTVDTVLEKNVTVTHSVNLGSGLRMVTGLRNIPQRETRGLFGAIAGFIEGGWTGMVDG
WYGYHHQNEQGSGYAADQKSTQNAINGITNMVNSVIEKMGSGGSGTDLAELLVLLLNERTLDFHDSNVKNLYEKVKSQLKNNAKEIGNGCF
EFYHKCNNECMESVKNGTYDYPKYSEESKLNREKIDPGSNLVTAFSNMDDMLQKAHLVIEGTFIYLRDSTEFFIRVRDGWKKLQLGELIPIPA
GSGAHIVMVDAYKPTGSGHHHHHH*

Certified by: Yinjia Zhu **Date:** 03/31/2023
