# Supplementary material for: Computational Design and Expression of Headless Influenza Hemagglutinin Antigens Toward a Modular Universal Nanoparticle Vaccine
Source: Antibodies (Basel). 2026 Jul 27;15(4):64. doi: 10.3390/antib15040064 (PMC13510027; doi:10.3390/antib15040064)
Supplement: Supplementary file 1 [file antibodies-15-00064-s001.zip › gels_hplc.zip!/H1-C15ZST_Protein_COA.pdf]

Certificate of Analysis

**Name:**	H1VRCC15ZST
**Type:**	Recombinant protein
**Shipping Condition:**	Dry Ice
**Lot Number:**	U8530750G0-4/P9JA001
**Expression Host :**	Mammalian
**Purification:**	HisTrap FF crude

**Total Amount:**	4.41 mg. 1.00 ml/tube, 9 tubes
**Concentration:**	0.49 mg/ml(by A280)
**Purity:**	≥65%(SDS-PAGE under non-reducing condition) ;N/A(SEC-HPLC)
**Endotoxin:**	≤0.2 EU/mg
**Storage And Handling:**	Store at -80.0°C. Avoid repeated freezing and thawing
**Storage Buffer:**	PBS, pH 7.2

GenScript USA, Inc.
860 Centennial Ave. Piscataway, NJ 08854
Tel: 1-732-885-9188

SDS-PAGE:

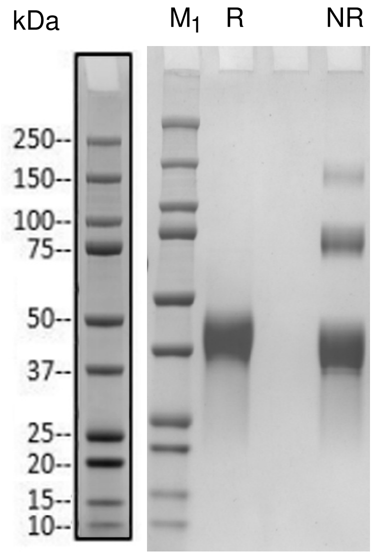

Lane M1: Protein Marker, Bio-rad, Cat. No. 1610374S, refer to annotated key on the left for size
R: Reducing condition
NR: Non-reducing condition

SEC-HPLC

Sample name: U8530750G0-1_After desalting
Instrument: 1260 Infinity II **Location:** P1-A8 **Injection volume:** 74.3
Column Name: Superdex 200 Increase 10/300GL **Column No:** 26#
Mobile Phase: 0.1M L-Arg in 2X PBS (pH 10.55)

—— Sample
.... Standard

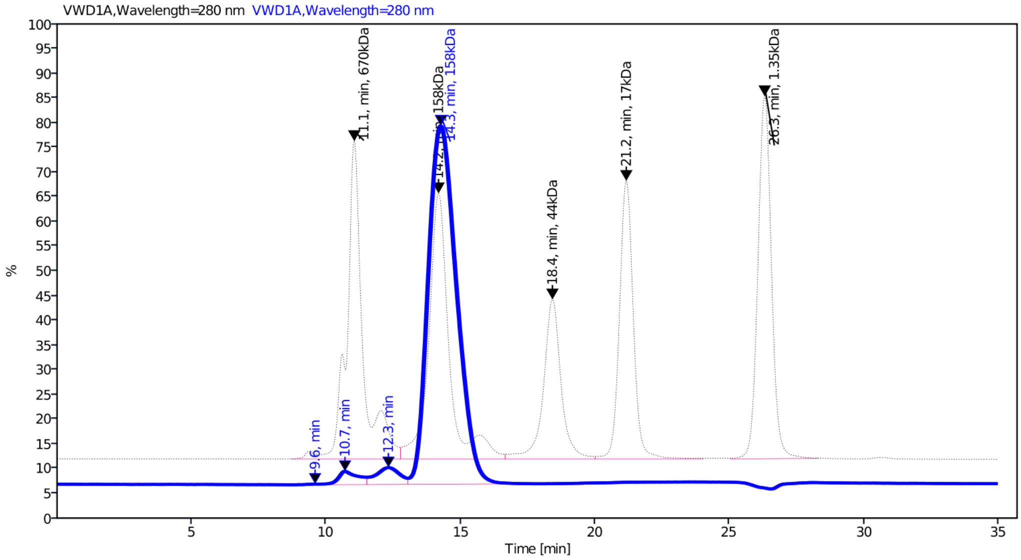

Signal: VWD1A,Wavelength=280 nm

RT [min]	Height	Width [min]	Area	Area%
9.6	0.1	0.8	2.0	0
10.7	1.4	1.9	75.1	2
12.3	1.8	1.5	112.5	4
14.3	38.7	4.6	2842.6	94
		Sum	3032.32	

GenScript
Make Research Easy

Appendix:

U8530750G0-1_H1VRCC15ZST

Protein Length=299 aa His_6: 294-299

**MGWSCIILFLVATATGVHSDTICIGYHANNSTDTVDTVLEKNVTVTHSVNLGSGLRMVTGLRNIPQRETRGLFGAIAGFIEGG
WTGMVDGWYGYHHQNEQGSGYAADQKSTQNAINGITNMVNSVIEKMGSGGSGTDLAELLVLLLNERTLDFHDSNVK
NLYEKVKSQLKNNAKEIGNGCFEFYHKCNNECMESVKNGTYDYPKYSEESKLNREKIDPGSASSTLLRFVSVQLMQQ
QVFVQDGSLAFITDSSKLYLKVPRGWREVQLGVLLESYPGSGAHIVMVDAYKPTGSGHHHHHH***

Certified by: Zhangling Wang **Date:** 01/08/2024
