# Supplementary material for: Computational Design and Expression of Headless Influenza Hemagglutinin Antigens Toward a Modular Universal Nanoparticle Vaccine
Source: Antibodies (Basel). 2026 Jul 27;15(4):64. doi: 10.3390/antib15040064 (PMC13510027; doi:10.3390/antib15040064)
Supplement: Supplementary file 1 [file antibodies-15-00064-s001.zip › gels_hplc.zip!/H2-C15ST_Protein_COA.pdf]

GenScript
Make Research Easy

Certificate of Analysis

**Name:**	H24HLZC15ST
**Type:**	Immune Recombinant Protein
**Shipping Condition:**	Dry Ice
**Lot Number:**	U760JXPQG0-14/P7IG001
**Expression Host :**	Mammalian
**Purification:**	AmMagTM Ni Magnetic Beads

**Total Amount:**	4.08 mg. 4.00 ml/tube, 3 tubes
**Concentration:**	0.34 mg/ml(by A280)
**Purity:**	≥40%(SDS-PAGE under non-reducing condition)
**Endotoxin:**	≤0.2 EU/mg
**Storage And Handling:**	Store at -80°C.Avoid repeated freezing and thawing
**Storage Buffer:**	PBS, pH 7.2

SDS-PAGE:

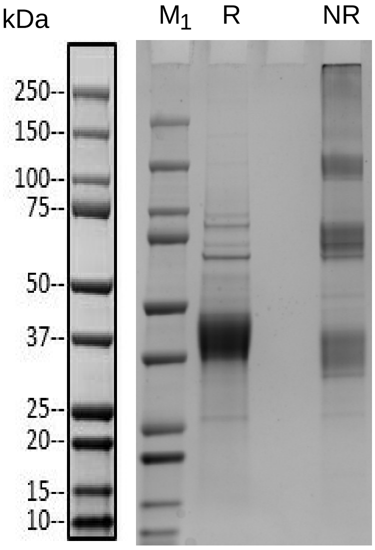

Lane M1: Protein Marker, Bio-rad, Cat. No. 1610374S, refer to annotated key on the left for size
R: Reducing condition
NR: Non-reducing condition

SEC-HPLC

Sample name: U760JXPQG0-11_after dialysis
Instrument: GR20010025 Agilent 1260 **Location:** 34
Column Name: TSKgel G3000SWxl **Column No:** 193#
Mobile Phase: 0.1 mol/L Na2SO4 in 0.118 mol/L Phosphate Buffer(pH 6.7±0.3)

Injection volume: 54.0
—— Sample
.... Standard

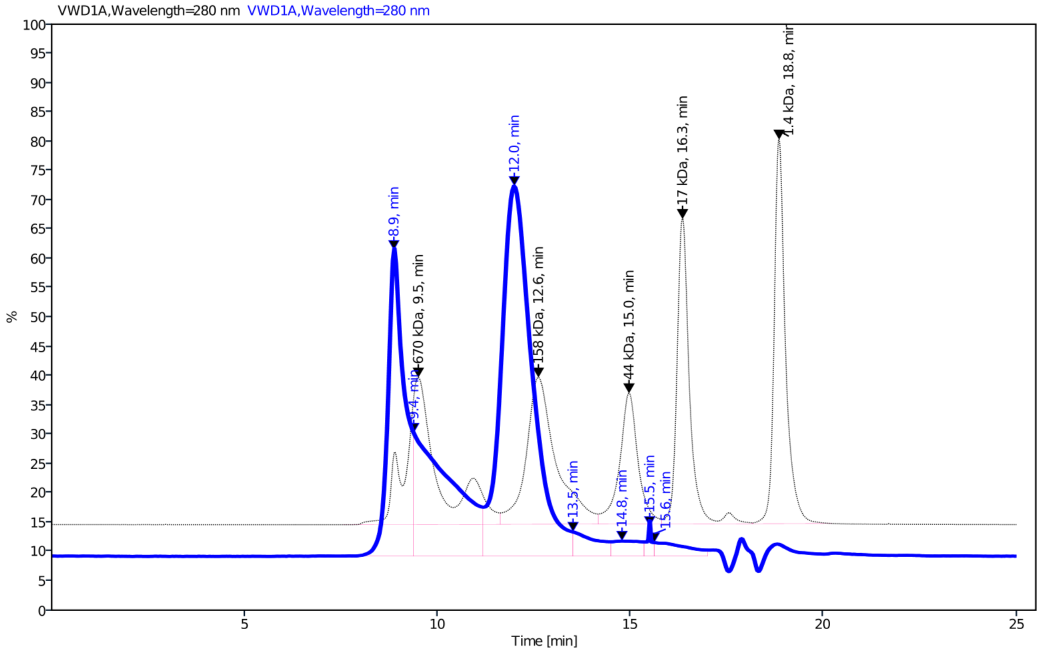

Signal: VWD1A,Wavelength=280 nm

RT [min]	Height	Width [min]	Area	Area%
8.9	12.0	0.5	360.0	22
9.4	4.8	1.2	336.0	21
12.0	14.5	2.3	817.0	50
13.5	0.9	1.0	40.6	2
14.8	0.6	0.9	29.7	2
15.5	1.2	0.3	10.7	1
15.6	0.5	1.4	31.7	2
		Sum	1625.67	

GenScript USA, Inc.
860 Centennial Ave. Piscataway, NJ 08854
Tel: 1-732-885-9188

Appendix:

>U760JXPQG0-11_H24HLZC15ST

Protein sequence: 300aa

MGWSCIILFLVATATGVHSRGDQICIGYHANNSTEKVDTILERNVTVTHAKDIGSGLVLATGLRNVPQIESRGLFGAIAGFIEGGWQGMVDG
WYGYHHSNDQGSGYAADKESTQKAFDGITNKVNSVIEKMGSGGSGTDLAELLVLMLNERTLDFHDSNVKNLYDKVRMQLRDNVKELGNG
CFEFYHKCDDECMNSVKNGTYDYPKYEEESKLNRNEIKPGSNLVTAFSNMDDMLQKAHLVIEGTFIYLRDSTEFFIRVRDGWKKLQLGELIPI
PAGSGAHIVMVDAYKPTGSGHHHHHH*

Certified by: Yujie Liu **Date:** 07/18/2023
