# Supplementary material for: Computational Design and Expression of Headless Influenza Hemagglutinin Antigens Toward a Modular Universal Nanoparticle Vaccine
Source: Antibodies (Basel). 2026 Jul 27;15(4):64. doi: 10.3390/antib15040064 (PMC13510027; doi:10.3390/antib15040064)
Supplement: Supplementary file 1 [file antibodies-15-00064-s001.zip › gels_hplc.zip!/H3-C15ST_Protein_COA.pdf]

Certificate of Analysis

**Name:**	H3VRCC15ST
**Type:**	Recombinant protein of immune products
**Shipping Condition:**	Dry Ice
**Lot Number:**	U4592IC100-4/P7ID001
**Expression Host :**	Mammalian
**Purification:**	HisTrap™ FF Crude

**Total Amount:**	500 µg. 1.25 ml/tube, 1 tube
**Concentration:**	0.40 mg/ml(by A280)
**Purity:**	≥70%(SDS-PAGE under non-reducing condition)
**Endotoxin:**	≤0.2 EU/mg
**Storage And Handling:**	Store at -80°C.Avoid repeated freezing and thawing
**Storage Buffer:**	PBS, pH 7.2

GenScript USA, Inc.
860 Centennial Ave. Piscataway, NJ 08854
Tel: 1-732-885-9188

SDS-PAGE:

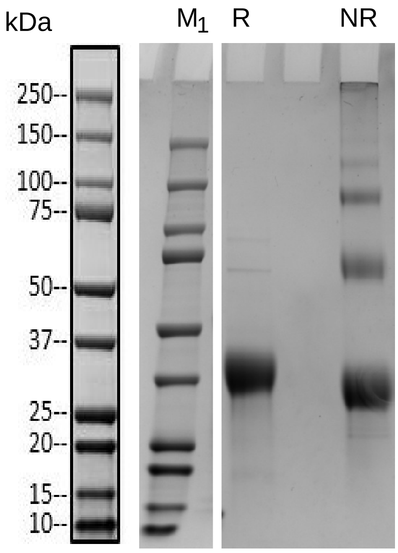

Lane M_1: Protein Marker, Bio-rad, Cat. No. 1610374S, refer to annotated key on the left for size
R: Reducing condition
NR: Non-reducing condition

SEC-HPLC

Sample name: U4592IC100-1_HPLC
Instrument: GR17010610 **Location:** P1-A-6 **Injection volume:** 49.0
Column Name: TSKgel G3000SWxl **Column No:** 172#
Mobile Phase: 0.1 mol/L Na2SO4 in 0.1 mol/L Phosphate Buffer(pH 6.7±0.3)

—— Sample
.... Standard

VWD1A,Wavelength=280 nm VWD1A,Wavelength=280 nm

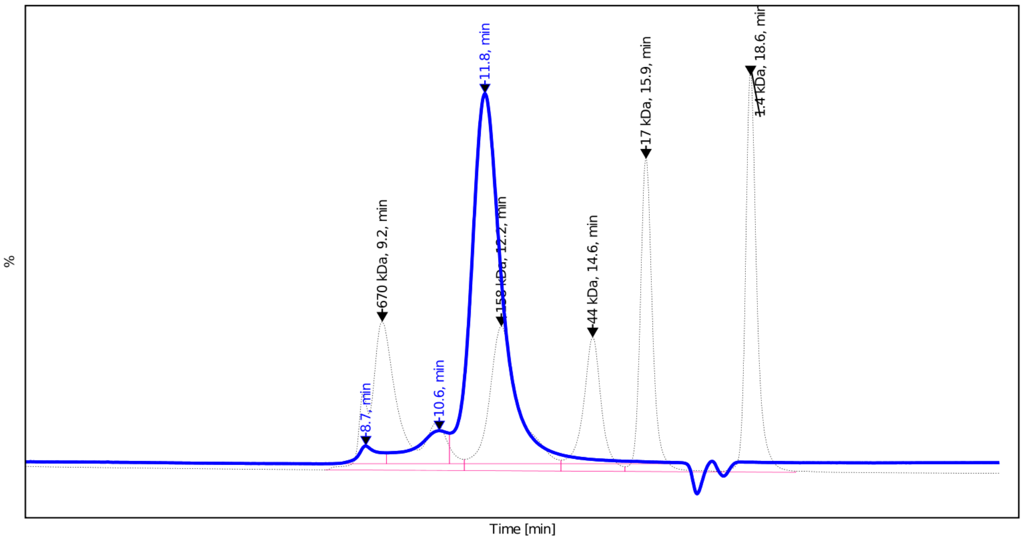

Signal: VWD1A,Wavelength=280 nm

RT [min]	Height	Width [min]	Area	Area%
8.7	1.0	1.3	37.8	3
10.6	1.9	1.6	115.1	8
11.8	21.6	5.8	1232.6	89
		Sum	1385.48	

GenScript USA, Inc.
860 Centennial Ave. Piscataway, NJ 08854
Tel: 1-732-885-9188

Appendix:

U4592IC100-1_H3VRCC15ST

Protein sequence: 305aa

MGWSCIILFLVATATGVHSSTATLCLGHHAVPNGTIVKTITNDQIEVTNATELVFPGCGVLKLATGMRNVPELKQTRGIFGAIAGFIENGWEG
MVDGWYGFRHQNSEGIGQAADLKSTQAAINQINGMVNRVIELMEQGGPDCYLAELLVALLNQHVIDLTDSEMRKLFERTKKQLRENAEDM
GNGCFKIYHKCDNACIGSIRNGTYDHDVYRDEALNNRFQIKSGGNLVTAFSNMDDMLQKAHLVIEGTFIYLRDSTEFFIRVRDGWKKLQLGE
LIPIPAGSGAHIVMVDAYKPTGSGHHHHHH*

Certified by: Zhongling Wang **Date:** 04/11/2023

GenScript USA, Inc.
860 Centennial Ave. Piscataway, NJ 08854
Tel: 1-732-885-9188
