# Supplementary material for: Computational Design and Expression of Headless Influenza Hemagglutinin Antigens Toward a Modular Universal Nanoparticle Vaccine
Source: Antibodies (Basel). 2026 Jul 27;15(4):64. doi: 10.3390/antib15040064 (PMC13510027; doi:10.3390/antib15040064)
Supplement: Supplementary file 1 [file antibodies-15-00064-s001.zip › gels_hplc.zip!/H3-Protein_COA.pdf]

GenScript
Make Research Easy

Certificate of Analysis

**Name:**	H3N2_C
**Type:**	Recombinant protein of immune products
**Shipping Condition:**	Dry Ice
**Lot Number:**	U9697HL070-4/P7IA001
**Expression Host :**	Mammalian
**Purification:**	HisTrap™ FF Crude

**Total Amount:**	9.80 mg. 1.00 ml/tube, 14 tubes
**Concentration:**	0.70 mg/ml(by A280)
**Purity:**	Too low to be detected(<30%)(SDS-PAGE under non-reducing condition)
**Endotoxin:**	≤0.1 EU/mg
**Storage And Handling:**	Store at -80°C.Avoid repeated freezing and thawing
**Storage Buffer:**	PBS, pH 7.2

GenScript USA, Inc.
860 Centennial Ave. Piscataway, NJ 08854
Tel: 1-732-885-9188

SDS-PAGE:

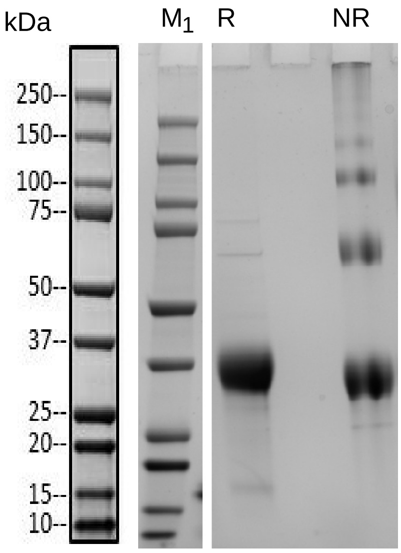

Lane M1: Protein Marker, Bio-rad, Cat. No. 1610374S, refer to annotated key on the left for size
R: Reducing condition
NR: Non-reducing condition

SEC-HPLC

Sample name: U9697HL070-1_after desalting

Instrument: GR16010687 **Location:** P2-B1 **Injection volume:** 43.0

Column Name: TSKgel G3000SWxl **Column No:** 159#

Mobile Phase: 0.1 mol/L Na2SO4 in 0.1 mol/L Phosphate Buffer(pH 6.7±0.3)

—— Sample

.... Standard

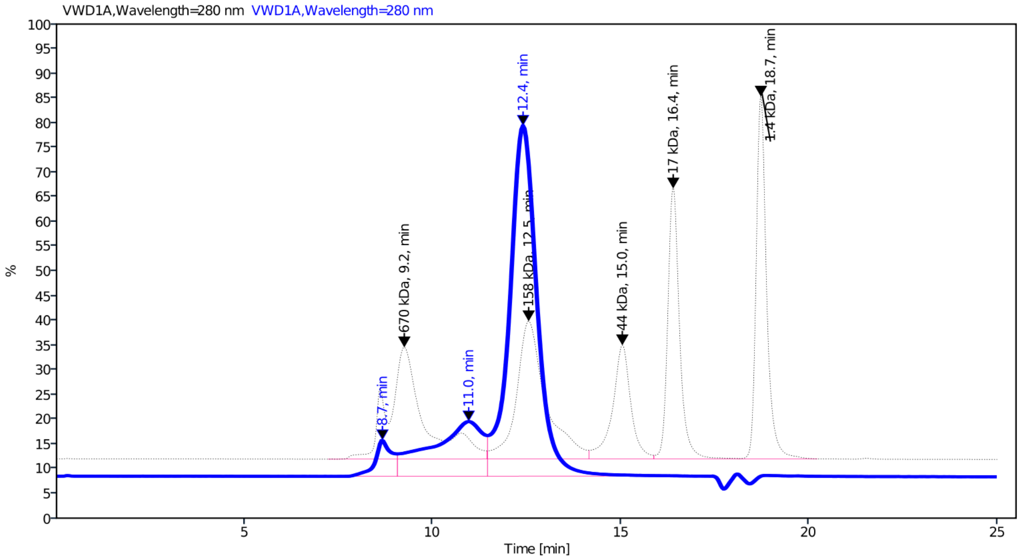

Signal: VWD1A,Wavelength=280 nm

RT [min]	Height	Width [min]	Area	Area%
8.7	3.2	1.3	98.8	5
11.0	4.8	2.4	456.9	21
12.4	30.9	4.7	1575.0	74
		Sum	2130.72	

GenScript USA, Inc.
860 Centennial Ave. Piscataway, NJ 08854
Tel: 1-732-885-9188

Appendix:

U9697HL070-1_H3N2_C

Protein sequence: 287aa

MGWSCIILFLVATATGVHSSTATLCLGHHAVPNGTIVKTITNDQIEVTNATELVFPGCGVLKLATGMRNVPELKQTRGIFGAIAGFIENGWEG
MVDGWYGFRHQNSEGIGQAADLKSTQAAINQINGMVNRVIELMEQGGPDCYLAELLVALLNQHVIDLTDSEMRKLFERTKKQLRENAEDM
GNGCFKIYHKCDNACIGSIRNGTYDHDVYRDEALNNRFQIKSGGNLVTAFSNMDDMLQKAHLVIEGTFIYLRDSTEFFIRVRDGWKKLQLGE
LIPIPAHHHHHH*

Certified by: Zhongling Wang **Date:** 01/06/2023

GenScript USA, Inc.
860 Centennial Ave. Piscataway, NJ 08854
Tel: 1-732-885-9188
