# Supplementary material for: Computational Design and Expression of Headless Influenza Hemagglutinin Antigens Toward a Modular Universal Nanoparticle Vaccine
Source: Antibodies (Basel). 2026 Jul 27;15(4):64. doi: 10.3390/antib15040064 (PMC13510027; doi:10.3390/antib15040064)
Supplement: Supplementary file 1 [file antibodies-15-00064-s001.zip › gels_hplc.zip!/H5V1-C15ST_Protein_COA.pdf]

GenScript
Make Research Easy

Certificate of Analysis

**Name:**	H5V14junC15ST
**Type:**	Recombinant protein of immune products
**Shipping Condition:**	Dry Ice
**Lot Number:**	U9007992G0-4/P7IE001
**Expression Host :**	Mammalian
**Purification:**	HisTrap™ FF Crude

**Total Amount:**	8.87 mg. 3.40 ml/tube, 3 tubes
**Concentration:**	0.87 mg/ml(by A280)
**Purity:**	≥30%(SDS-PAGE under non-reducing condition)
**Endotoxin:**	≤0.2 EU/mg
**Storage And Handling:**	Store at -80°C.Avoid repeated freezing and thawing
**Storage Buffer:**	PBS, pH 7.2

SDS-PAGE:

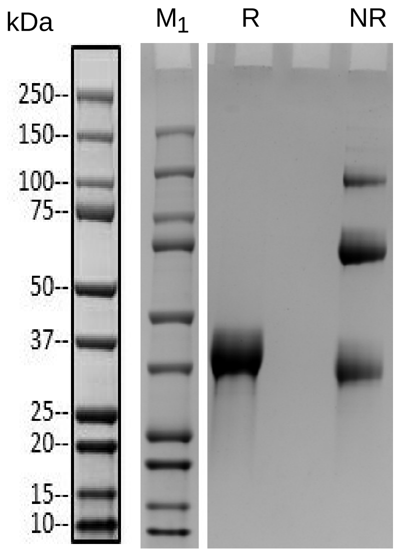

Lane M1: Protein Marker, Bio-rad, Cat. No. 1610374S, refer to annotated key on the left for size
R: Reducing condition
NR: Non-reducing condition

SEC-HPLC

Sample name: U9007992G0-1_after desalting
Instrument: GR16010687 Agilent 1260 **Location:** P1-B3 **Injection volume:** 22.0
Column Name: TSKgel G3000SWxl **Column No:** 183#
Mobile Phase: 0.1 mol/L Na2SO4 in 0.118 mol/L Phosphate Buffer(pH 6.7±0.3)

—— Sample
········ Standard

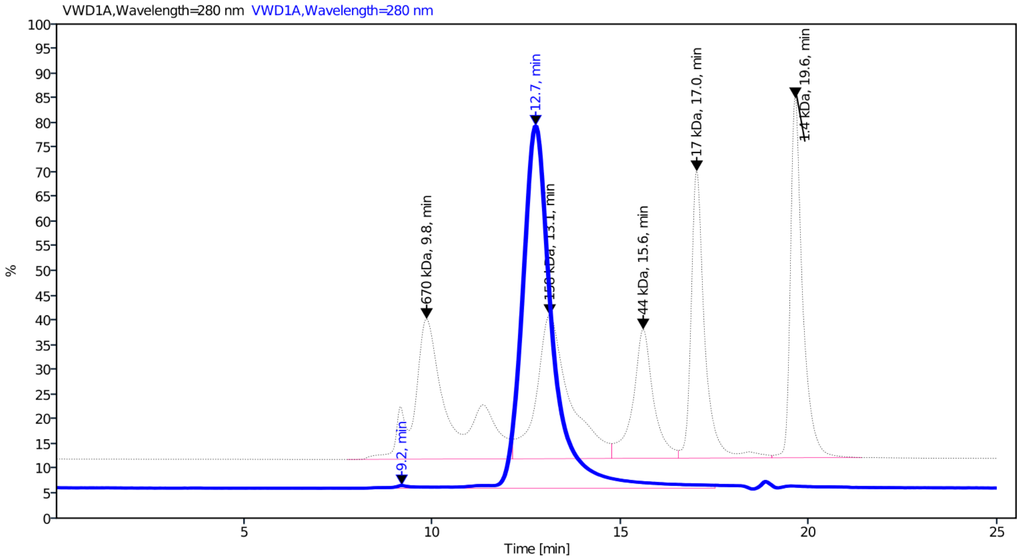

Signal: VWD1A,Wavelength=280 nm

RT [min]	Height	Width [min]	Area	Area%
9.2	0.2	1.9	10.9	1
12.7	31.4	6.8	1777.6	>99
		Sum	1788.57	

GenScript USA, Inc.
860 Centennial Ave. Piscataway, NJ 08854
Tel: 1-732-885-9188

GenScript
Make Research Easy

Appendix:

U9007992G0-1_H5V14junC15ST

Protein sequence: 300aa

MGWSCIILFLVATATGVHSKSDQICIGYHANNSTEQVDTIMEKNVTVTHAQDIGSGLVLATGLRNSPQRETRGLFGAIAGFIEGGWQGMVD
GWYGYHHSNEQGSGYAADKESTQKAIDGVTNMVNSIIDKMGSGGSGTDLAELLVLMLNERTLDFHDSNVKNLYDRVRLQLRDNAKELGNG
CFEFYHKCDNECMESVRNGTYDYPQYSEEARLKREEISPGSNLVTAFSNMDDMLQKAHLVIEGTFIYLRDSTEFFIRVRDGWKKLQLGELIPI
PAGSGAHIVMVDAYKPTGSGHHHHHH*

Certified by: Yujie Lin **Date:** 05/31/2023

GenScript USA, Inc.
860 Centennial Ave. Piscataway, NJ 08854
Tel: 1-732-885-9188
