# Supplementary material for: Computational Design and Expression of Headless Influenza Hemagglutinin Antigens Toward a Modular Universal Nanoparticle Vaccine
Source: Antibodies (Basel). 2026 Jul 27;15(4):64. doi: 10.3390/antib15040064 (PMC13510027; doi:10.3390/antib15040064)
Supplement: Supplementary file 1 [file antibodies-15-00064-s001.zip › gels_hplc.zip!/H6-C15ST_Protein_COA.pdf]

Certificate of Analysis

**Name:**	H65BNYC15ST
**Type:**	Immune Recombinant Protein
**Shipping Condition:**	Dry Ice
**Lot Number:**	U760JXPQG0-24/P7IG001
**Expression Host :**	Mammalian
**Purification:**	AmMagTM Ni Magnetic Beads

**Total Amount:**	4.03 mg. 1.00 ml/tube, 1 tube; 4.00 ml/tube, 3 tubes
**Concentration:**	0.31 mg/ml(by A280)
**Purity:**	N/A
**Endotoxin:**	≤0.2 EU/mg
**Storage And Handling:**	Store at -80°C.Avoid repeated freezing and thawing
**Storage Buffer:**	PBS, pH 7.2

GenScript USA, Inc.
860 Centennial Ave. Piscataway, NJ 08854
Tel: 1-732-885-9188

SDS-PAGE:

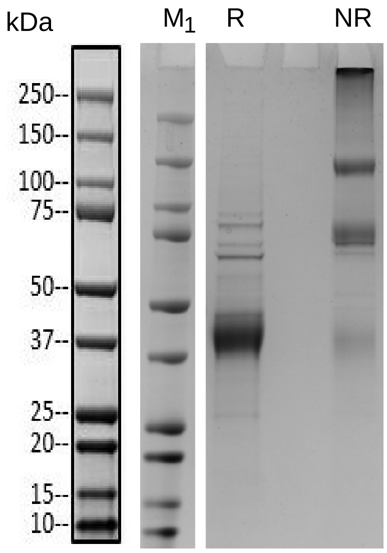

Lane M1: Protein Marker, Bio-rad, Cat. No. 1610374S, refer to annotated key on the left for size
R: Reducing condition
NR: Non-reducing condition

SEC-HPLC

Sample name: U760JXPQG0-21_after dialysis
Instrument: GR20010025 Agilent 1260 **Location:** 33
Column Name: TSKgel G3000SWxl **Column No:** 193#
Mobile Phase: 0.1 mol/L Na2SO4 in 0.118 mol/L Phosphate Buffer(pH 6.7±0.3)

Injection volume: 63.0
—— Sample
.... Standard

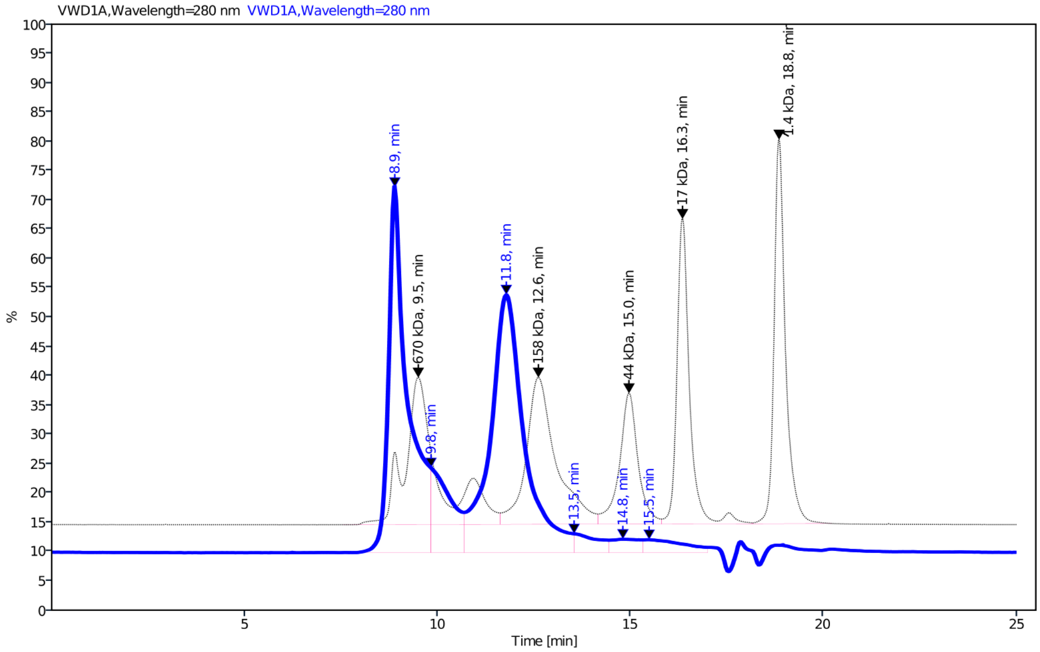

Signal: VWD1A,Wavelength=280 nm

RT [min]	Height	Width [min]	Area	Area%
8.9	18.4	1.9	643.7	37
9.8	4.3	0.9	155.8	9
11.8	13.0	2.9	803.6	47
13.5	0.9	0.9	40.8	2
14.8	0.7	0.9	34.4	2
15.5	0.7	1.7	48.2	3
		Sum	1726.49	

GenScript USA, Inc.
860 Centennial Ave. Piscataway, NJ 08854
Tel: 1-732-885-9188

GenScript
Make Research Easy

Appendix:

>U760JXPQG0-21_H65BNYC15ST

Protein sequence: 300aa

MGWSCIILFLVATATGVHSKSDKICIGYHANNSTTKVDTILEKNVTVTHSVELGSGLRLATGLRNVPQIETRGLFGAIAGFIEGGWTGMIDGW
YGYHHENSQGSGYAADKESTQKAIDGITNKVNSIIDKMGSGGSGTDLAELLVLLLNERTLDLHDANVKNLHEKVRSQLRDNANDLGNGCFE
FWHKCNNECMESVKNGTYDYPKYQKESRLNRQKIEPGSNLVTAFSNMDDMLQKAHLVIEGTFIYLRDSTEFFIRVRDGWKKLQLGELIPIPA
GSGAHIVMVDAYKPTGSGHHHHHH*

Certified by: Zhongling Wang **Date:** 07/18/2023

GenScript USA, Inc.
860 Centennial Ave. Piscataway, NJ 08854
Tel: 1-732-885-9188
